# Dissecting super-enhancer driven transcriptional dependencies reveals novel therapeutic strategies and targets for group 3 subtype medulloblastoma

**DOI:** 10.1186/s13046-022-02506-y

**Published:** 2022-10-22

**Authors:** Meng Li, Yujie Han, Chaochen Wang, Wenfeng Kang, Wenyan Jiang, Lei Zhang, Yujie Tang

**Affiliations:** 1 Key Laboratory of Cell Differentiation and Apoptosis of National Ministry of Education, Department of Pathophysiology, Shanghai Jiao Tong University School of Medicine, 280 South Chongqing Road, 200025 Shanghai, People’s Republic of China; 2 Department of Breast Surgery, The Second Affiliated Hospital, Zhejiang University School of Medicine, Zhejiang University, Hangzhou, People’s Republic of China; 3 ZJU-UoE Institute, Zhejiang University School of Medicine, International Campus, Zhejiang University, Haining, People’s Republic of China; 4 Shanghai Key Laboratory of Reproductive Medicine, Department of Histoembryology, Genetics and Developmental Biology, Shanghai Jiao Tong University School of Medicine, 280 South Chongqing Road, Shanghai, 200025 People’s Republic of China

**Keywords:** Group 3 subtype medulloblastoma, Super enhancer, Transcriptional dependencies of cancer, Novel therapeutic strategies and targets, *ARL4D*

## Abstract

**Background:**

Medulloblastoma is the most common malignant pediatric brain tumor and group 3 subtype medulloblastoma (G3-MB) exhibits the worst prognosis. Super enhancers (SEs) are large clusters of enhancers that play important roles in cancer through transcriptional control of cell identity genes, oncogenes and tumor-dependent genes. Dissecting SE-driven transcriptional dependencies of cancer leads to identification of novel oncogenic mechanisms, therapeutic strategies and targets.

**Methods:**

Integrative SE analyses of primary tissues and patient-derived tumor cell lines of G3-MB were performed to extract the conserved SE-associated gene signatures and their oncogenic potentials were evaluated by gene expression, tumor-dependency and patient prognosis analyses. SE-associated subtype-specific upregulated tumor-dependent genes, which were revealed as members of SE-driven core transcriptional regulatory network of G3-MB, were then subjected to functional validation and mechanistic investigation. SE-associated therapeutic potential was further explored by genetic or pharmaceutical targeting of SE complex components or SE-associated subtype-specific upregulated tumor-dependent genes individually or in combination, and the underlying therapeutic mechanisms were also examined.

**Results:**

The identified conserved SE-associated transcripts of G3-MB tissues and cell lines were enriched of subtype-specifically upregulated tumor-dependent genes and MB patients harboring enrichment of those transcripts exhibited worse prognosis. Fourteen such conserved SE-associated G3-MB-specific upregulated tumor-dependent genes were identified to be members of SE-driven core transcriptional regulatory network of G3-MB, including three well-recognized TFs (*MYC*, *OTX2* and *CRX*) and eleven newly identified downstream effector genes (*ARL4D*, *AUTS2*, *BMF*, *IGF2BP3*, *KIF21B*, *KLHL29*, *LRP8*, *MARS1*, *PSMB5*, *SDK2* and *SSBP3*). An OTX2-SE-*ARL4D* regulatory axis was further revealed to represent a subtype-specific tumor dependency and therapeutic target of G3-MB via contributing to maintaining cell cycle progression and inhibiting neural differentiation of tumor cells. Moreover, BET inhibition with CDK7 inhibition or proteasome inhibition, two combinatory strategies of targeting SE complex components (*BRD4*, *CDK7*) or SE-associated effector gene (*PSMB5*), were shown to exhibit synergistic therapeutic effects against G3-MB via stronger suppression of SE-associated transcription or higher induction of ER stress, respectively.

**Conclusions:**

Our study verifies the oncogenic role and therapeutic potential of SE-driven transcriptional dependencies of G3-MB, resulting in better understanding of its tumor biology and identification of novel SE-associated therapeutic strategies and targets.

**Supplementary Information:**

The online version contains supplementary material available at 10.1186/s13046-022-02506-y.

## Background

Medulloblastoma (MB) is the most common malignant pediatric brain tumor and one of the leading causes of brain-tumor patient death of children. Current MB treatment includes surgical resection followed by radiation and intensive chemotherapy. The establishment of a consensus molecular subtyping standard is a milestone of developing targeted MB therapy [1]. There are four major subtypes of MB: WNT, SHH, Group 3 and Group 4, which carry distinct gene expression profiles, epigenetic landscapes, genetic mutations and clinical outcomes [2]. Among the four subtypes, group 3 subtype MB (G3-MB) exhibits the worst prognosis as they tend to carry amplification of *MYC*, to metastasis and to relapse following therapy [3]. Therefore, patients of G3-MB need more effective therapy most urgently.

Super-enhancers (SEs) are large proximal clusters of enhancers with extraordinary enrichment of H3K27Ac, transcription factors (TFs) and coactivators [4, 5]. They exert oncogenic functions via driving transcription of cell identity genes, oncogenes and tumor-dependent genes in cancer cells [4, 5]. Those genes can be categorized into upstream TFs and downstream effector genes, which together comprise SE-driven core transcriptional regulatory network [5, 6]. SE-associated TFs often self-regulate and mutually regulate the others, thus forming cross-regulated feed-forward loops called SE-driven core regulatory circuitry [5, 6]. Dissecting SE-driven transcriptional dependency not only helps better understanding the cellular origin and oncogenic mechanisms of cancer, but also facilitate identification of novel therapeutic strategies or targets. Targeting BRD4, a crucial component of the SE complex, with BET inhibitor (BETi) has been shown to effectively suppress SE-associated transcription and growth of many cancers in preclinical tests [7]. Moreover, SE-associated malignancy genes are often found to be more vulnerable to CDK7 inhibition, which targets the general transcription factor TFIIH, an integral component of the RNA polymerase II pre-initiation complex. CDK7 inhibitor (CDK7i) is found to exhibit selective suppression on cancer cells via preferentially targeting SE-driven transcriptional addiction [7]. More importantly, BETi and CDK7i drugs have already entered human clinical trials for cancer therapy. Targeting SE complex suppresses transcription of members of SE-associated core transcriptional regulatory network preferentially and effectively [8, 9]. This is extremely helpful for treating tumor types highly addicted to oncogenic master TFs, which are often difficult to be directly targeted by small-molecule inhibitors. Alternatively, some SE-associated downstream tumor-dependent effector genes could serve as promising drug targets for developing novel cancer therapy [10, 11].

There has been some progress in unveiling SE’s oncogenic functions and the underlying molecular mechanisms in G3-MB. A study has reported the SE landscape of all four subtypes of MB based on epigenetic profiling of human tumor tissues, which reinforces the inter-subgroup tumor heterogeneity of MB via analyzing SE-driven core regulatory circuitry [12]. As expected, *MYC* and *OTX2*, the two well-established oncogenic driver TFs of G3-MB, are revealed as subtype-specific SE-associated oncogenes of G3-MB tumor tissues. Moreover, another study has reported CRX and NRL as another two SE-associated subtype-specific tumor-dependent TFs. They are shown to be master regulators of the photoreceptor transcriptional program that represents a G3-MB specific tumor dependency [13]. Furthermore, both BETi and CDK7i have been reported to effectively treat pre-clinical models of G3-MB [14–17], but their impacts on SE’s oncogenic functions have not been evaluated yet. Notably, it has been shown that the enhancer landscape of primary tissues of G3-MB exhibited poor overlap and correlation with those of tumor cell lines [12], therefore, whether the commonly used patient-derived primary G3-MB lines could serve as proper models for further investigating oncogenic functions and therapeutic potential of SE-associated transcription remains to be determined. In this study, we aimed to perform integrative SE analyses of primary tissues and patient-derived tumor cell lines of G3-MB to verify the oncogenic role of SE-driven transcriptional dependencies and further explore their therapeutic potential in preclinical models of G3-MB.

## Methods

### Cell culture

293T cell line was obtained from Cell Bank of Chinese Academy of Science (Shanghai, China). D425, MB002, HD-MB03 and UW228 cell lines were kindly provided by Prof. Yoon-jae Cho (Oregon Health & Science University). D425, UW228 and 293T were cultured in DMEM (BI-01–052-1ACS, Biological Industries) supplemented with 10% FBS (F2442, Sigma). MB002 and HD-MB03 were cultured in Tumor Stem Media (TSM) as previously described [16]. Drosophila S2 cell line was cultured in Schneider’s Insect Medium (S0146, Sigma) supplemented with 10% heat-inactivated FBS (S711-001S, Lonsera) in humidified air at 37 °C (Forma Reach-In CO_2_ Incubator, Modal 3951, Thermo Fisher Scientific).

### Plasmid construction, lentivirus packaging and infection

ShRNAs and cas13d-sgRNA were cloned into pLKO.1-puro vector and pLentiRNACRISPR_005-hU6-DR_BsmBI-EFS-RfxCas13d-NLS-2A-Puro-WPRE (Addgene plasmid #138147) vector, respectively.

Lentivirus was generated by co-transfection of 293T cells with above mentioned plasmids and packaging plasmids pMD2.G (Addgene plasmid # 12259) and psPAX2 (Addgene plasmid # 12260). Lentiviral particles were concentrated via PEG method and resuspended in PBS for infection.

Cells were infected with indicated lentivirus at multiplicity of infection (MOI) of 1 ~ 5 for two days and subjected to puromycin selection for another three days. Then the cells were harvested and subjected to FACS analyses of cell proliferation, cell apoptosis and cell cycle, or seeded into 96-well plate in triplicate (5000 cells per well) for cell viability tests.

All shRNA and cas13d-sgRNA sequences were listed in supplementary Table 1.

### Compounds

THZ1 (HY-80013), JQ1 (HY-13030), Marizomib (HY-10985) were purchased from MedChem Express (NJ, USA).

### Immunoblot assay

Whole cell lysates were obtained by lysing cells with RIPA buffer supplemented with Protease Inhibitor Cocktail Set III (539134, Calbiochem) and Phosphatase Inhibitor Cocktail 3 (P0044, Sigma). Protein concentration was determined with Pierce BCA Protein Assay (23225, Thermo Fisher Scientific). Equal amount of protein was loaded for immunoblot analysis. Antibodies used for immunostaining were listed in supplementary Table 2.

### RNA extraction, reverse transcription and quantitative real-time PCR (RT-qPCR)

Total RNA was extracted using TRI Reagent (TR118, MRC) according to the manufacturer’s instructions. Reverse transcription (RT) was performed with High Capacity cDNA Reverse Transcription Kit (4368813, Thermo Fisher scientific). Quantitative real-time PCR (qPCR) analysis was performed with Fast Real-time PCR System (ABI, 7900HT) using FastStart Universal SYBR Green Master (ROX) (04913850001, Roche). Total cDNA of Drosophila S2 cells, serving as spike-in reagent, was added to total cDNA with mass ratio of 1:10. RT-qPCR assays were performed in triplicates and the data are presented as mean ± SD (standard deviation). The qPCR primers were listed in supplementary Table 3.

### Cell viability, CI, proliferation, apoptosis, and cell cycle assays

For cell viability measurement, cells were seeded into 96-well plates (5000 cells per well) and exposed to drug treatment or not. The viabilities of the seeded wells were then measured by Celltiter-Glo (G9243, Promega). Cell viability assays were performed in triplicates and the data are presented as the means ± SD. For synergistic investigation, the combination index (CI) was calculated with CompuSyn software (ComboSyn, Inc.). FACS analyses of cell proliferation, cell apoptosis and cell cycle were performed with Click-iT EdU Alexa Fluor 647 Flow Cytometry Assay Kit (C10640, Invitrogen), Annexin V-FITC Apoptosis Detection Kit (556547, BD Biosciences), Cell cycle staining kit (CCS012, Multi Science), respectively. FACS data were acquired from BD Fortessa (BD Biosciences) or CytoFLEX (Beckman Coulter) FACS instrument and analyzed with Flowjo software (FlowJo, LLC).

### Extreme limiting dilution assay (ELDA)

MB002 or D425 cells were infected with lentiviruses expressing shSCR, shARL4D-1 or shARL4D-2 for two days followed by puromycin selection for another three days. Then the cells were digested and seeded in 96-well plate at increasing numbers from 1 cell/well (*n* = 30), 10 cells/well (*n* = 10), 20 cells/well (*n* = 10), 30 cells/well (*n* = 10), 40 cells/well (*n* = 10), 50 cells/well (*n* = 10), 100 cells/well (*n* = 8), 250 cells/well (*n* = 8). Cells were allowed to grow for two weeks, and the number of wells containing tumor spheres were counted manually under the light microscope. Published ELDA software (http://bioinf.wehi.edu.au/software/elda/) or L-Calc™ software (https://www.stemcell.com/l-calc-software.html#section-data-and-publications) was used to calculate the frequency of tumorsphere forming cells under each condition.

### ChIP-qPCR

Chromatin immunoprecipitation (ChIP) coupled with qPCR (ChIP-qPCR) was performed as described previously [18]. Briefly, cells were fixed by 1% formaldehyde for 8 min at room temperature (RT) with rotation, quenched by 0.125 M glycine. The cells were digested by MNase (NEB, M0247S) and followed by sonication for 5 cycles (20 s on/30 s off for one cycle). Then the chromatin was incubated with indicated primary antibodies (H3K27Ac, Active Motif #39133, or OTX2, ProteinTech #13497–1-AP) with rotation overnight at 4 °C. The antibody-chromatin complex was immunoprecipitated with magic beads (26162, Thermo Fischer Scientific) with rotation at 4 °C for 4 h. Then the immunoprecipitated DNA was extracted followed by qPCR. ChIP-qPCR results of indicated primary antibodies were calculated by normalization to ChIP-INPUT. ChIP-qPCR assays were performed in triplicates and the data are presented as mean ± SD. The ChIP-qPCR primers were listed in Supplementary table 4.

### Chromosome conformation capture coupled with PCR (3C-PCR)

The 3C-PCR procedure was performed as previous described [19] with slight modification. Briefly, Cells were fixed by 1% formaldehyde for 8 min at RT with rotation, quenched by 0.125 M glycine. The crosslinked cells were incubated with ice-cold lysis buffer (10 mM Tris–HCl, pH8.0; 10 mM NaCl; 0.2% NP-40; 1 × Protease inhibitors, Roche) at a concentration 1.5 × 10^7^ cells/500 μL with rotation at 4 °C for 30 min. Nuclei were harvested, washed with ice-cold lysis buffer once, resuspended with 200 μL 0.5% SDS and incubated at 62 °C for 10 min. Then, 570 μL water and 100 μL 10% TritonX-100 was added to the sample and incubated at 37 °C for 15 min to sequester the SDS. Ten percent volume of the sample was saved as Sample 1 (S1). Chromatin sample was digested overnight by 375 U of restriction enzyme HindIII-HF (NEB, R3104T) or MboI (NEB, R0147M) with rotation at 37 °C, which was then heat inactivated at 80 °C or 62 °C for 20 min, respectively. Ten percent volume of the digested sample was saved as Sample 2 (S2). Remaining sample was incubated with ligation solution [1 × NEB T4 DNA ligase buffer with 10 mM ATP (NEB, B0202); 1% TritonX-100; 100 μg/mL BSA; 4000 U T4 DNA Ligase (NEB, M0202)] at RT for 4 h with rotation. Ten percent volume of the ligated sample was saved as Sample 3 (S3). DNA was then extracted by phenol chloroform isoamyl alcohol (25:24:1) followed by PCR.

Promoter-located constant and SE-located test 3C-PCR primers were designed for detecting DNA loop-structure in gene loci of *ARL4D* and *PSMB5*. Primers were named after location, initial of gene symbol (HindIII-digestion related) and also restriction enzyme in the case of MboI.

The 3C-PCR primers were listed in Supplementary table 5.

### Pooled-sgRNA CRISPR interference

Anneals of sgRNA oligos targeting the same SE region were pooled, cloned into lentiGuide-puro (Addgene #52963). Pooled sgRNA plasmids were extracted by FastPure Plasmid Mini Kit (Vazyme, DC201-01) and packaged into lentivirus as previously described. Stable dCas9-KRAB-expressing cells were infected with the pooled-sgRNA lentivirus and qPCR-tested for transcription interference on SE-associated gene. All CRISPRi-sgRNA sequences were listed in supplementary Table 6.

### G3-MB tumor xenograft

All in vivo experimental procedures were approved by the Animal Care and Use Committee of Shanghai Jiao Tong University School of Medicine and performed according to the guidelines. For orthotopic inoculation, each 8-10 weeks old female nude mice (BALB/c^nu/nu^) (Lingchang, Shanghai) were injected with 7.5 × 10^4^ MB002 cells with stably expressing GFP and firefly luciferase proteins (MB002-GFP-luc) (suspended in 3 μl PBS). Cells were stereotactically injected into each nude mouse’s cerebellum 2.1 mm below the dura at a location 2 mm right of the midline and 2 mm posterior of the bregma. Then the tumor burden of the mice was monitored by in vivo imaging system (IVIS). The mice were intraperitoneally injected with D-luciferin (75 mg/kg, P1043, Promega) and were imaged by the Xenogen IVIS200 Imaging System (Perkin-Elmer). The signal of the total bioluminescence flux intensity (p/s) for each xenografted nude mouse was collected to represent tumor burden. The IVIS signal data are presented as mean ± SEM.

### In vivo drug treatment

The orthotopic xenograft models were randomly divided into 4 groups, and treated with vehicle, Marizomib (150 μg/kg, tail vein injection, once a week), JQ1 (50 mg/kg, intraperitoneal injection, twice a week) or in combination, respectively.

### RNA-seq and ChIP-seq

D425 was treated with 0.1 μM THZ1 for 6 h or 1 μM JQ1 for 24 h, lysed in Trizol and sent to the company (Smartquerier Biomedicine, Shanghai, China) for RNA sequencing. For ChIP sequencing, D425, MB002 and HD-MB03 cells were harvested, fixed by 1% formaldehyde, snap-frozen and sent to the company (Romics, Shanghai, China) together with H3K27Ac antibody (AM39133, Active Motif).

### RNA-seq data processing

RNA-seq data were mapped to the cDNA sequences of GRCh38 by Salmon [20]. Mapped read counts were normalized using DESeq2 [21] followed by differential gene expression analysis.

### ChIP-seq data processing

All ChIP-seq data sets were aligned to the human genome (build version: GRCh38/hg38) using Bowtie 2 (version 2.3.0) [22]. SAM files generated by Bowtie2 were then converted to BAM files with samtools (version 1.9) [23]. Multi-mappers and duplicates were filtered out by sambamba (version 0.7.1) [24]. ChIP-seq peaks over input sample were identified using a peak-finding algorithm, MACS2 (version 2.2.6) [25]. A *q* value of 0.05 was set as threshold of enrichment for all data sets. Active enhancers were defined as regions of ChIP-seq enrichment for the enhancer-associated histone modification H3K27Ac outside of promoters (excluding the ± 2.5 kb region flanking the promoter). In order to accurately capture dense clusters of enhancers, stitching distance of 12.5 kb was allowed for separate H3K27Ac regions. Super-enhancers were identified and analyzed as described previously [26].

### Gene Set Variation Analysis (GSVA) and Gene Set Enrichment Analysis (GSEA)

Gene Set Variation Analysis [27] (GSVA) were performed on the data from indicated public database using GSVA package in R. Gene Set Enrichment Analysis (GSEA) was performed according to the instructions on the website (http://www.broadinstitute.org/gsea/index.jsp) as previously described [28].

### Data source

Gene expression and survival data were obtained from R2 platform (http://r2.amc.nl). Human MB patient or normal cerebellum gene expression datasets: Cavalli (Tumor Medulloblastoma-Cavalli-763-rma_sketch-hugene11t), Pomeroy (Mixed Medulloblastoma public-Pomeroy-204-MAS5.0-u133a), Pfister (Tumor Medulloblastoma-Pfister-167-fpkm-mb500rs1), U133P2 (Tumor Medulloblastoma-Pfister-223-MAS5.0-u133p2, Tumor Medulloblastoma-Gilbertson-76-MAS5.0-u133p2, Normal cerebellum-Roth-9-MAS5.0-u133p2). Patient survival dataset: Cavalli (Tumor Medulloblastoma-Cavalli-763-rma_sketch-hugene11t).

H3K27Ac ChIP-seq data of D283 and D341 lines were acquired from GSE92585.

CERES gene effect scores for evaluating tumor-dependency were from DepMap Public 20Q2 Achilles_gene_effect on DepMap platform (https://depmap.org/portal/). For Tumor dependency analysis, a CERES score of -0.1 was selected as cutoff instead of what is being normally used, -0.5, so that some of the well-described oncogenes of G3-MB, such as *CRX* and *NRL*, would not be mis-identified to be dispensable based on their CERES scores in the tested G3-MB lines.

### Statistical analyses

GraphPad Prism 6.0 software was applied for the statistical analysis. Significance was calculated by two-tailed Student’s t test for data with two groups and One-way ANOVA for data with more than two groups. Two-way ANOVA was used to compare IVIS bioluminescence flux intensity. The statistical significance of Kaplan-Meier survival curves was determined by Log-rank (Mantel-Cox) test. The FDR value of GSEA was generated by GSEA software. *p < 0.05, **p < 0.01, ***p < 0.001, ****p < 0.0001.

## Results

### Characterization of SE-associated gene signatures of patient-derived primary G3-MB lines

**Fig. 1:**
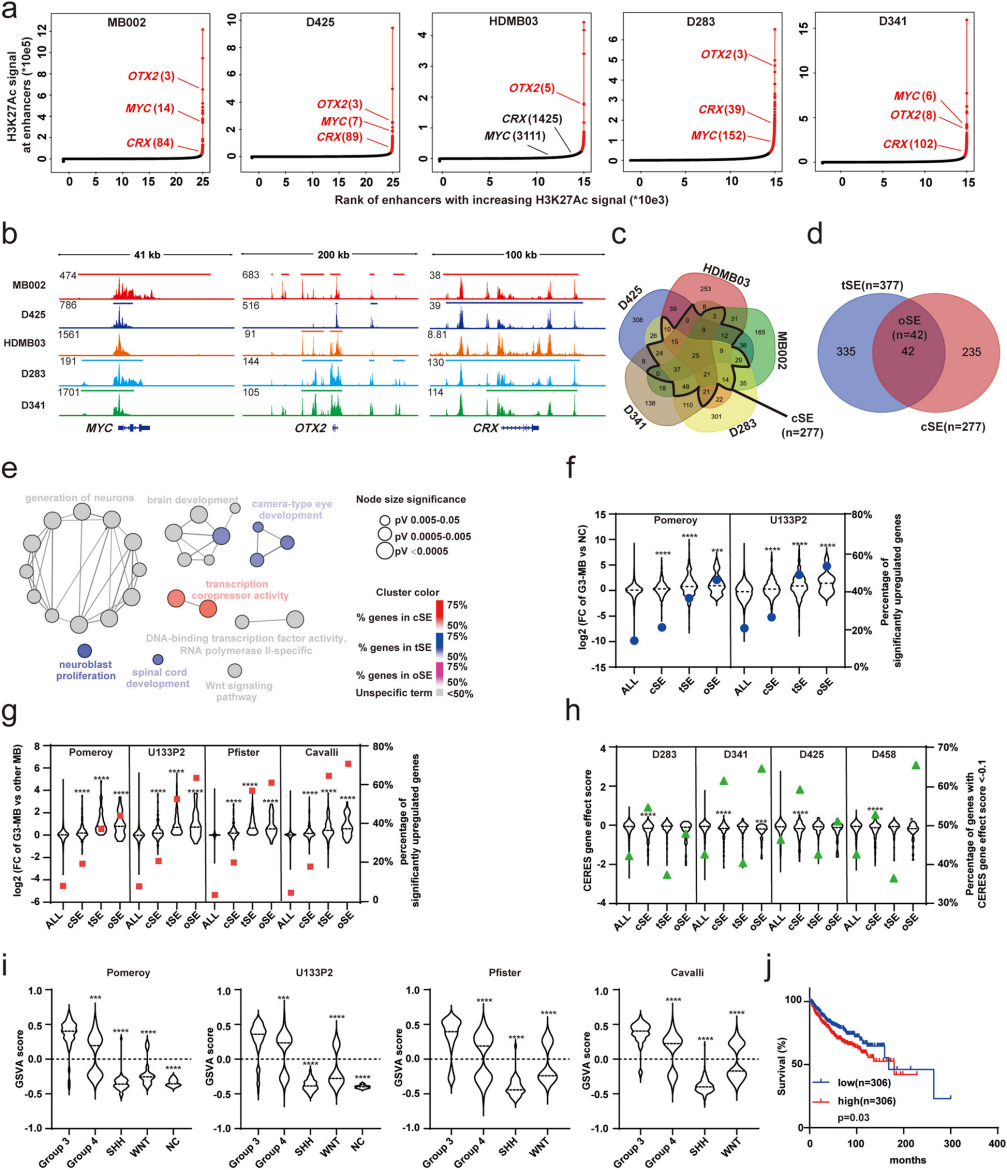
Characterization of SE-associated gene signatures of patient-derived primary G3-MB lines. **a** Enhancer profile of five G3-MB lines based on H3K27Ac ChIP-seq signal in reads per million per base pair (rpm/bp). Enhancers are ranked by increasing H3K27Ac ChIP-seq signal. Super enhancers (SEs) are highlighted in red with ranks of selected SE-associated genes. **b** Gene tracks of H3K27Ac ChIP-seq signal across five G3-MB lines at SE regions near *MYC*, *OTX* and *CRX*. SE regions are depicted in colored lines over the gene tracks. **c** Venn diagram analysis of SE-associated genes from the five G3-MB cell lines. **d** Venn diagram showing overlapping SE-associated genes (oSE) of tSE and cSE. **e** Gene ontology (GO) analyses of genes of cSE, tSE and oSE. The biological process (BP) cluster comparation analysis was performed with Cytoscape plug-in ClueGo. **f-g** Violin plots showing log2_FC (fold change) of gene expression (left Y axis) or dot plots showing percentage of significantly upregulated genes (right Y axis) for genes in all, cSE, tSE and oSE categories, when comparing G3-MB tissues with normal cerebellum (NC) in **f** (log2_FC > 1, FDR < 0.05) or the other three MB subtypes in **g** ( log2_FC > 0.5, FDR < 0.05) in the indicated MB datasets. **h** Violin plots showing CERES gene effect score (left Y axis) or dot plots of percentage of genes with CERES gene effect score < -0.1 (right Y axis) in all, cSE, tSE and oSE categories in the indicated G3-MB lines. **i** Violin plots showing GSVA score of oSE genes in four MB subgroups or NC of the indicated MB datasets. **j** Kaplan-Meier survival analysis of the GSVA scores of oSE genes in Cavalli dataset of MB. The patient cohort was stratified as high versus low groups based on median GSVA score. Statistical significance was determined by one-way ANOVA (**f-i**) or two-sided log-rank test (**j**), respectively

To characterize the SE landscape of patient-derived primary G3-MB lines, we performed chromatin-immunoprecipitation with sequencing (ChIP-seq) of H3K27Ac antibody and RNA-seq analyses in three human primary G3-MB cell lines (MB002, D425, HD-MB03). Previously published H3K27Ac ChIP-seq and RNA-seq data of two other G3-MB lines (D283 and D341) were also obtained for SE profiling [29]. ROSE (Rank Ordering of Super-Enhancers) algorithm was used for calling SE and SE-associated genes. As shown in Fig. 1a-b, *MYC*, *OTX2* and *CRX* were found to be within the top-rank SE-associated genes of G3-MB cell lines as previously reported in primary G3-MB tissues [12, 13]. We defined SE-associated genes recurrently identified in at least three G3-MB lines as “cellular_SE-associated_gene_signature” (cSE) (Fig. 1c). We also extracted SE-associated genes of G3-MB tissues from a previously published study [12] as “tissue_SE-associated_gene_signature” (tSE). The 42 genes shared between cSE and tSE were defined as “overlapping_SE-associated_gene_signature” (oSE) (Fig. 1d). Gene ontology (GO) analyses revealed they were all significantly enriched in biological processes related to nervous system development and transcription regulation (Fig. 1e).

Next, we examined the oncogenic potential of the three SE-associated gene signatures of G3-MB. The “ALL” gene signature, which contained all measured genes in each dataset, was used as control. For gene expression analyses, four MB tissue transcriptomic datasets (Pomeroy [30], U133P2 [31–33], Pfister [31], Cavalli [34]) were obtained from R2 website with two of them (Pomeroy, U133P2) containing normal cerebellum control data. Compared to ALL, all the three SE-associated gene signatures are enriched of significantly upregulated genes of G3-MB versus normal cerebellum (NC) or the other three MB subgroups, and the oSE exhibits the highest enrichment (Fig. 1f-g). For gene dependency analyses, CERES gene effect scores of four G3-MB lines (D283, D341, D425 and D458) calculated based on whole-genome CRISPR-Cas9 screening results were obtained from DepMap Public 20Q2 [35]. We found cSE and oSE but not tSE were enriched of tumor-dependent genes in all four G3-MB lines (Fig. 1h). To delineate the impact of SE-associated transcription of G3-MB on clinical outcome, we performed gene set variation analysis (GSVA) of cSE, tSE and oSE in the MB tissue transcriptomic datasets and found they are all significantly enriched in G3-MB versus NC or the other subgroups (Fig. 1i and S1a-b). Moreover, MB patients harboring higher enrichment of these SE-associated gene signatures consistently exhibit inferior survival (Fig. 1j and S1c-d). Together, these data demonstrated the conserved SE-associated transcripts between primary tumor cell lines and tissues of G3-MB were enriched of subtype-specific upregulated tumor-dependent genes and MB patients harboring enrichment of those transcripts exhibited worse prognosis, indicating these G3-MB lines could be used for further exploring the therapeutic potential of SE-associated transcription.

### Establishment of SE-driven core transcriptional regulatory network of G3-MB

**Fig. 2:**
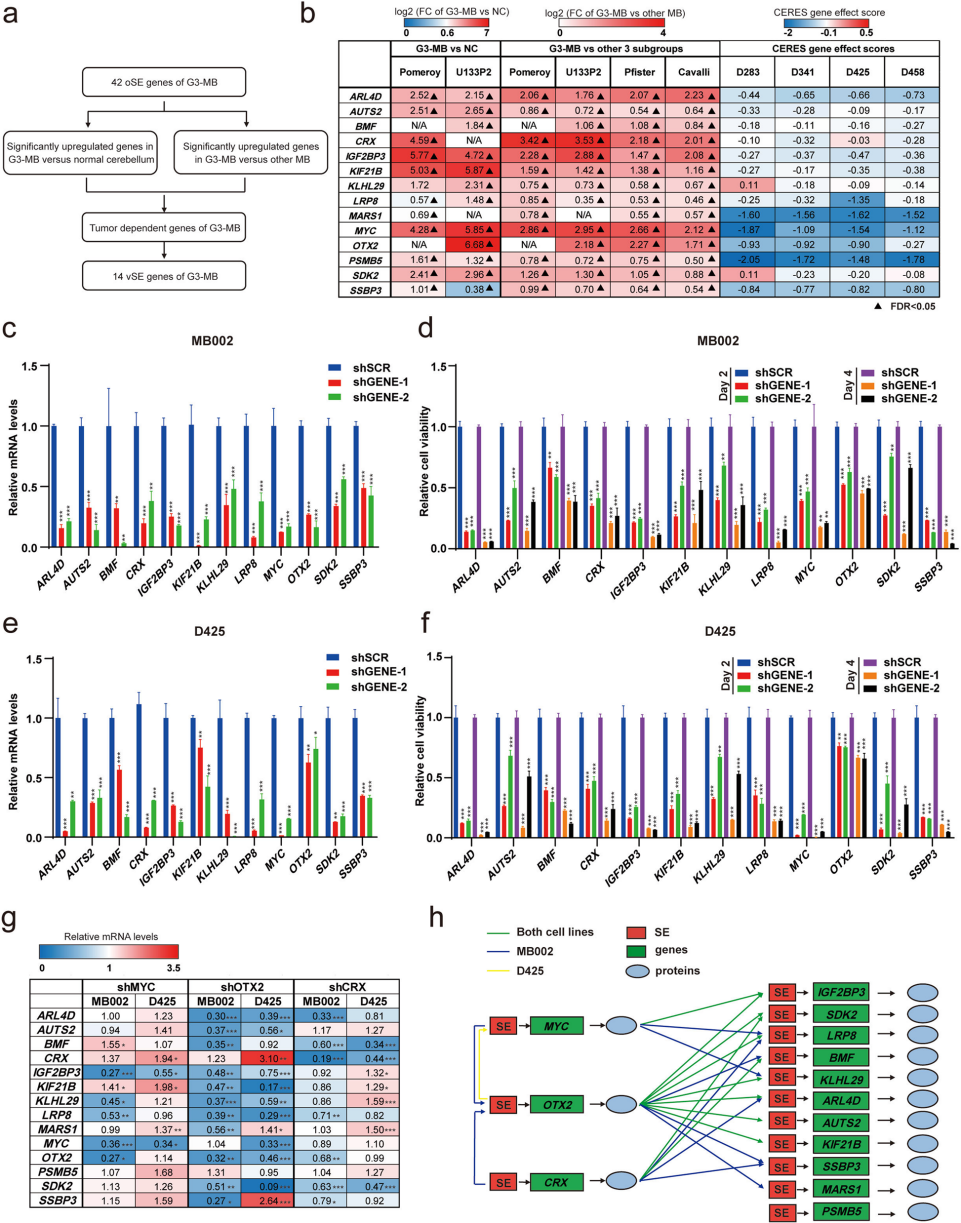
Establishment of SE-driven core transcriptional regulatory network of G3-MB. **a** Workflow of identifying vital SE-associated gene signature (vSE) of G3-MB. **b** Summary of the results from the gene expression and tumor dependency analyses of the 14 vSE genes identified in **a**. **c-f** RT-qPCR analysis of the 12 selected vSE genes in MB002 (**c**) or D425 (**e**) cells with each of these genes knocked down by two separate shRNAs individually. Cell viability of MB002 (**d**) or D425 (**f**) cells under above-mentioned conditions were measured at Day 0/2/4 post puromycin selection. **g** RT-qPCR analysis of vSE genes in MB002 or D425 cells when *MYC*, *OTX2* and *CRX* were knocked down by shRNA individually. The mean relative expression levels are shown. **h** Schematic diagram of the SE-associated TF-effector-gene regulatory axis in the identified SE-driven core transcriptional regulatory network of G3-MB. All RT-qPCR and cell viability assays were performed in triplicate and the data are presented as mean ± SD. Statistical significance was determined by one-way ANOVA (**c-f**) and two-tailed unpaired *t* test (**g**), respectively

To decipher SE-associated subtype-specific oncogenic mechanisms of G3-MB, oSE genes were examined to identify members of the SE-driven core transcriptional regulatory network. The following criteria were utilized: (1) significantly upregulated in G3-MB versus NC (log2FC > 0.6, FDR < 0.05 in at least one dataset) or the other three MB subtypes (log2FC > 0.2, FDR < 0.05 in at least three datasets); (2) tumor-dependent (CERES score < -0.1 in at least two G3-MB lines). Fourteen such SE-associated genes were found to meet all these criteria and defined as “vital_SE-associated_gene_signature” (vSE), including the three well-established TFs (*MYC*, *OTX2* and *CRX*) and eleven newly identified downstream effector genes of G3-MB (*ARL4D*, *AUTS2*, *BMF*, *IGF2BP3*, *KIF21B*, *KLHL29*, *LRP8*, *MARS1*, *PSMB5*, *SDK2* and *SSBP3*) (Fig. 2a-b). Nine such effector genes were selected for tumor-dependency verification with RNA interference approach. *MYC*, *OTX2* and *CRX* were tested in parallel as positive controls. *MARS1* and *PSMB5* were exempted from such tests based on their extremely low CERES scores in all analyzed G3-MB lines (Fig. 2b). As shown in Fig. 2c-f, knocking down of these genes individually with two separate shRNAs all markedly dampened the growth of MB002 and D425 cells in vitro, supporting their tumor dependency in G3-MB. Next, we measured the impact of knockdown of *MYC*, *OTX2* or *CRX* individually on the transcript levels of the other thirteen vSE genes, to build up their regulatory connections within the SE-driven core transcriptional regulatory network of G3-MB (Fig. 2g-h and S2a-c). To be noted, we did not detect any consistent cross-regulated feed-forward loops of the three SE-associated TFs within the two G3-MB lines.

### BET inhibitor works synergistically with CDK7 inhibitor on suppressing SE-driven core transcriptional regulatory network of G3-MB

**Fig. 3:**
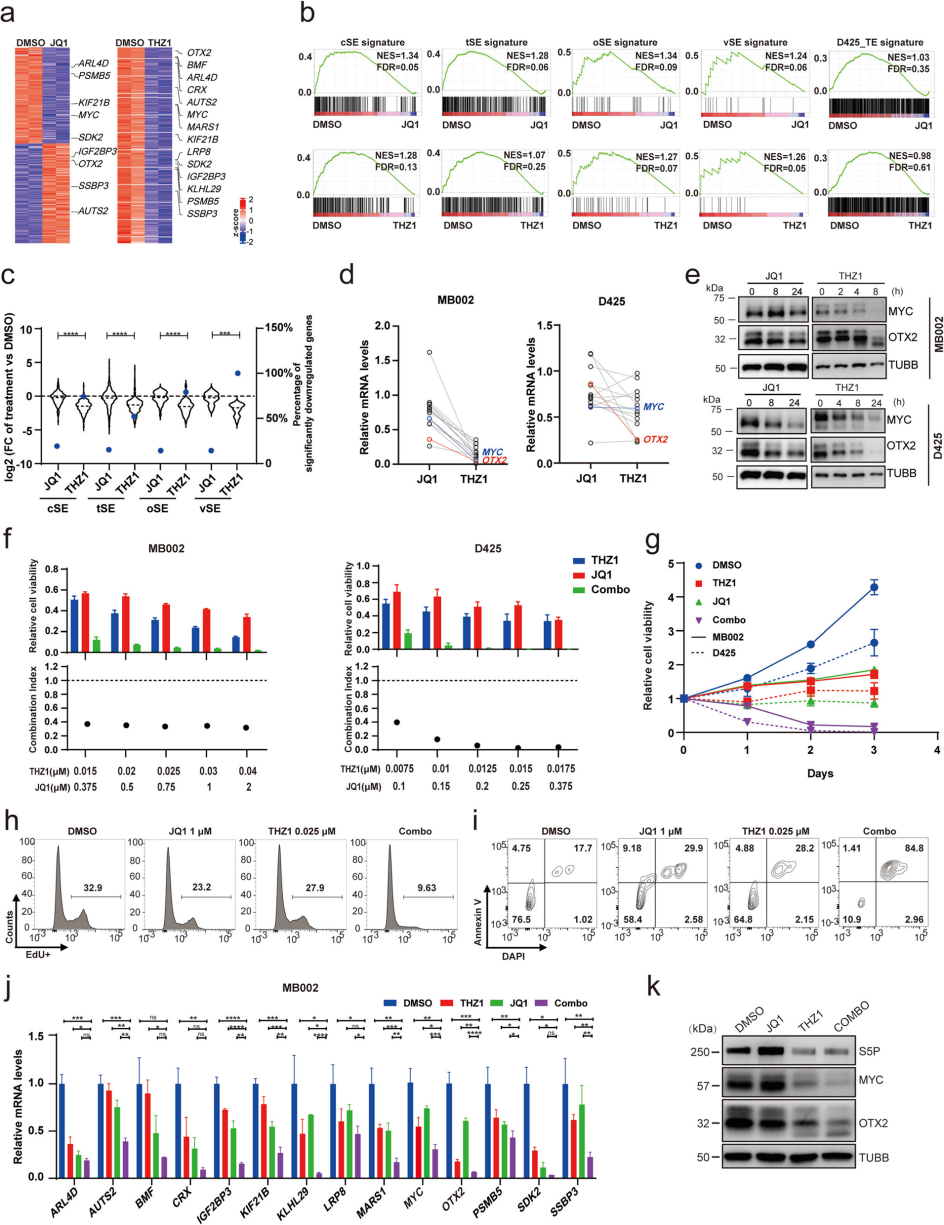
BET and CDK7 inhibitors synergistically suppresses SE-driven core transcriptional regulatory network of G3-MB. **a** Heatmap of significantly differential genes actively expressed (mean FPKM ≥ 1, adjusted *p*-value ≤ 0.05) in D425 treated with DMSO, 1 μM JQ1 for 24 h or 0.1 μM THZ1 for 6 h. vSE genes are highlighted. **b** Groupwise GSEA analysis of SE signatures as labeled in D425 treated with either JQ1 (upper panels) or THZ1 (lower panels) versus DMSO. D425_TE signature serves as control. **c** Plots on expression (left Y axis, violin) and percentage of significantly downregulated genes (log2_FC < -1, FDR < 0.05) (right Y axis, dot) of different SE signatures genes from D425 treated same as in **a**. **d** RT-qPCR analysis of vSE genes in MB002 and D425 treated with 1 μM JQ1 or 0.1 μM THZ1 for 6 h. *MYC* and *OTX2* are highlighted. **e** Immunoblot of MYC and OTX2 in MB002 and D425 treated with the same drugs and dosages as in (**d**) for the indicated time. **f** Cell viability and corresponding CI of MB002 and D425 cells treated with THZ1 and JQ1 at the indicated concentrations for 72 h. A CI value of less than 1 indicates synergy. **g** Day0-normalized cell viability of MB002 and D425 single- or combo-treated with THZ1 (12.5 or 15 nM for each cell line) and 0.5 μM JQ1. **h**-**i** FACS analyses on cell proliferation (**h**) and apoptosis (**i**) of MB002 cells with labeled treatments. **j** RT-qPCR analysis of vSE genes in MB002 cells single- or combo-treated with 1 μM JQ1 and 25 nM THZ1 for 6 h. **k** Immunoblot of MYC and OTX2 in MB002 cells treated with 0.5 μM JQ1 or 0.05 μM THZ1 for 8 h. RT-qPCR and cell viability assays were performed in triplicate and the data are presented as mean ± SD. Statistical significance is determined either by two-tailed paired *t* test (**c**) or one-way ANOVA (**j**)

Both BETi and CDK7i have been reported to effectively suppress growth of G3-MB in vitro and in vivo [14–17]. However, as well-recognized SE-targeted therapeutic strategies, their impacts on SE-associated transcription of G3-MB remain unexplored. To do so, we performed RNA-seq analyses of JQ1 (1 μM for 24 h) or THZ1 (0.1 μM for 6 h) treated D425 cells in parallel. As shown in Fig. 3a, THZ1 but not JQ1 induced remarkable genome-wide downregulation of active transcripts. Next, we examined how JQ1 or THZ1 affected SE-associated transcription in G3-MB cells. Gene set enrichment analysis (GSEA) results showed that both JQ1 and THZ1 could markedly suppress transcription of cSE, tSE, oSE or vSE signature (NES > 1, FDR ≤ 0.25). In contrast, they did not exhibit such significant inhibition on D425_TE signature, which is composed of bottom ranked 1099 typical enhancer (TE) associated genes (same number as SE-associated genes) of D425 cells (Fig. 3b). When we compared the inhibitory effects between THZ1 and JQ1 in treating D425 cells, we found all tested SE-associated gene signatures of G3-MB were more robustly downregulated by THZ1 than JQ1 (Fig. 3c). The stronger anti-SE activity of THZ1 versus JQ1 was further verified by RT-qPCR analysis of all fourteen vSE genes as well as immunoblot analysis of MYC and OTX2 proteins in both D425 and MB002 lines (Fig. 3d-e).

Notably, the combination of BETi and CDK7i have been shown before in other cancer types to exert their synergistic inhibitory effects via stronger suppression of SE-associated oncogenic transcriptional activity [8, 9, 11]. Accordingly, we tested the in vitro combinatory therapy of JQ1 and THZ1 against D425 and MB002 and found their combination exhibited synergistically inhibitory effects against both G3-MB lines as well (Fig. 3f). THZ1 + JQ1 was more potent in suppressing cell proliferation and inducing cell apoptosis, thus resulting in stronger cytocidal effects (Fig. 3g-i). Moreover, our RT-qPCR results showed their combination induced stronger transcriptional downregulation of all fourteen vSE genes (Fig. 3j). Their combinatory inhibition on *MYC* and *OTX2* at protein level was further confirmed by immunoblot analysis (Fig. 3k). Taken together, these results illustrated the inhibitory effects of BETi or CDK7i on SE-associated transcription individually and further revealed their therapeutic synergy against G3-MB cells via stronger suppression of SE-driven core transcriptional regulatory network, thus proving the therapeutic potential of treating G3-MB via targeting SE complex components.

### BET inhibitor works synergistically with proteasome inhibitor on suppressing G3-MB

**Fig. 4:**
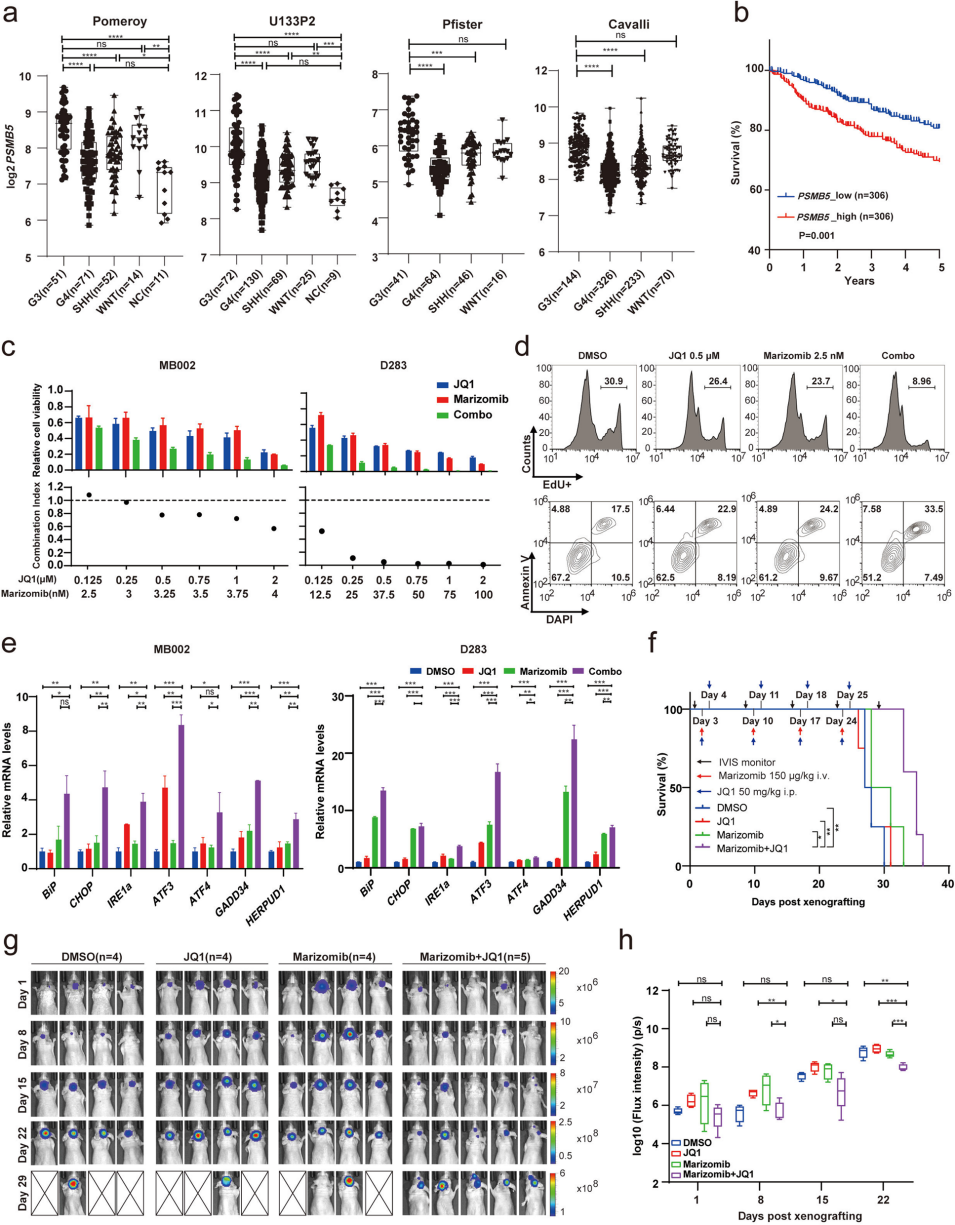
BET inhibitor works synergistically with proteasome inhibitor on suppressing G3-MB. **a** Box plots showing *PSMB5* mRNA levels of four MB subgroups or NC in the indicated MB datasets. **b** Kaplan-Meier analysis of the overall survival of MB patients stratified by *PSMB5* mRNA levels in Cavalli dataset. **c** Cell viability (top) and CI (bottom) of MB002 and D283 cells treated with JQ1 and Marizomib at the indicated concentrations for 72 h. **d** FACS analyses of cell proliferation (top) and apoptosis (bottom) of MB002 cells treated with Marizomib and JQ1 at the indicated concentrations. **e** RT-qPCR analysis of the selected ER stress genes in MB002 and D283 cells treated with JQ1 (1 μM for MB002, 2 μM for D283) and Marizomib (4 nM for MB002, 50 nM for D283) individually or in combination for 8 h. **f** Kaplan-Meier survival curve of nude mice carrying orthotopic xenografts of MB002-GFP-luc cells treated with JQ1 and Marizomib individually or in combination as indicated. **g-h** The tumor growth of xenografted nude mice treated as described in (**f**) was monitored by IVIS weekly. The collected mice images with corresponding signal scale bars measured in p/s are shown in **g**. Crossed white box indicates death of the treated mouse. Box plots showing the signals of total bioluminescence flux intensity for xenografted nude mice of each treatment condition and the data are presented as mean ± SEM in **h**. All RT-qPCR and cell viability assays were performed in triplicate and the data are presented as mean ± SD. Statistical significance was determined by one-way ANOVA (**a** and **e**), two-sided log-rank test (**f**) and two-way ANOVA (**h**), respectively

To further explore the therapeutic potential of SE-driven transcriptional dependencies in G3-MB, we evaluated the inhibitory effects of targeting SE complex components (*BRD4* or *CDK7*) in combination with targeting SE-associated tumor-dependent effector genes. Within the identified fourteen members of G3-MB’s SE-driven core transcriptional regulatory network, *PSMB5* is the only one having clinically available small-molecule inhibitors. It encodes a β subunit of 20S proteolytic core of the 26S proteasome complex [36], and has been shown to act as the direct target of various proteasome inhibitor (PSI) drugs including Bortezomib, Carfilzomib and Marizomib [37]. As shown in Fig. 4a-b, *PSMB5* is significantly upregulated in G3-MB and its higher expression is associated with worse prognosis of MB patients. Based on the alignment of its SE regions of across multiple G3-MB tissues and cell lines, D283 was selected as another G3-MB cell line model for *PSMB5* investigation (Fig.S3a-b). Meanwhile, UW228, a human non-G3 MB cell line, was used as a control for following SE analysis and validation. As shown in Fig.S3c-d, RNA-seq and ChIP-qPCR analyses validated the higher transcript levels of *PSMB5* and the stronger enrichment of H3K27Ac at the conserved proximal SE regions of *PSMB5* in multiple G3-MB lines versus UW228, respectively. We also performed 3C-PCR analysis and identified stronger chromatin looping and interaction between the SE region and the promoter region of *PSMB5* in G3-MB cells versus UW228 cells (Fig.S3e-f). Moreover, CRISPR interference (CRISPRi) silencing of *PSMB5*'s SE region resulted in significant downregulation of its transcript level and cell viabilities of G3-MB cells (Fig S3g-h). Together, these results proved the crucial role of *PSMB5*’s SE in regulating its transcription in G3-MB.

To be noted, in line with our findings in D425 and MB002 cells, *PSMB5* transcription was sensitive to BET inhibition or CDK7 inhibition, but not knockdown of *MYC*, *OTX2* or *CRX* in D283 cells (Fig.S3i-l). Then we also performed single-cell transcriptomic analysis of G3-MB tumor cells using single-cell RNA-seq (scRNA-seq) data of MB primary tissues from a recent study [38]. As shown in Fig.S4, G3-MB tumor cells were found to exhibit stronger *PSMB5* expression than tumor cells of the other three MB subtypes at single-cell level. Moreover, the tumor cell subpopulations expressing the highest level of *PSMB5* (GP3-B1) are different from the ones of *MYC*, *OTX2* or *CRX* (GP3-B2 for *MYC*, GP3-C2 for *OTX2* and *CRX*), further supporting the involvement of unidentified SE-associated TFs in regulating *PSMB5* transcription in G3-MB (Fig.S4).

Notably, PSI drug Marizomib has been previously reported to exhibit in vitro inhibitory activity against G3-MB or Group 4 subtype MB (G4-MB) alone or in combination with radiation [39]. It has also been proved to effectively penetrate the blood–brain barrier (BBB) and already entered human clinical trials for treating multiple brain cancers like DIPG and glioblastoma [40, 41]. Therefore, we chose Marizomib for further drug combination testing. We performed in vitro combinatory therapy tests on multiple G3-MB lines of Marizomib with JQ1 or THZ1. Synergy was detected between Marizomib and JQ1 but not THZ1 (Fig. 4c and S5a-b). Like THZ1 + JQ1, Marizomib + JQ1 was also more effective in suppressing cell proliferation, inducing cell apoptosis and generating cytocidal effects (Fig. 4d and S5c). To be noted, the anti-tumor synergy between BETi and PSI has been reported in other tumor types to result from stronger activation of ER stress and unfolded protein response (UPR) [42, 43]. As shown in Fig. 4e, our RT-qPCR results revealed Marizomib + JQ1 induced stronger expression of seven representative UPR genes (*BiP*, *CHOP*, *IRE1α*, *ATF3*, *ATF4*, *GADD34*, *HERPUD1*), indicating a similar synergistic mechanism in treating G3-MB. We further tested the combination therapy of JQ1 and Marizomib in an orthotopic xenograft model of G3-MB to demonstrate its in vivo therapeutic efficacy. Nude mice orthotopically implanted with MB002-GFP-luc cells were treated with JQ1 (50 mg/kg, intraperitoneal injection, twice a week), Marizomib (150 μg/kg, intravenous injection, once a week) or in combination. As shown in Fig. 4f-h, while treatment of JQ1 or Marizomib at such low dosage alone did not generate obvious therapeutic effect, their combination resulted in significantly slower tumor progression and longer survival of xenografted nude mice. None of these treatment conditions obviously affected mice bodyweight (Fig.S5d).

### ARL4D represents a novel subtype-specific tumor-dependency and therapeutic target of G3-MB

**Fig. 5:**
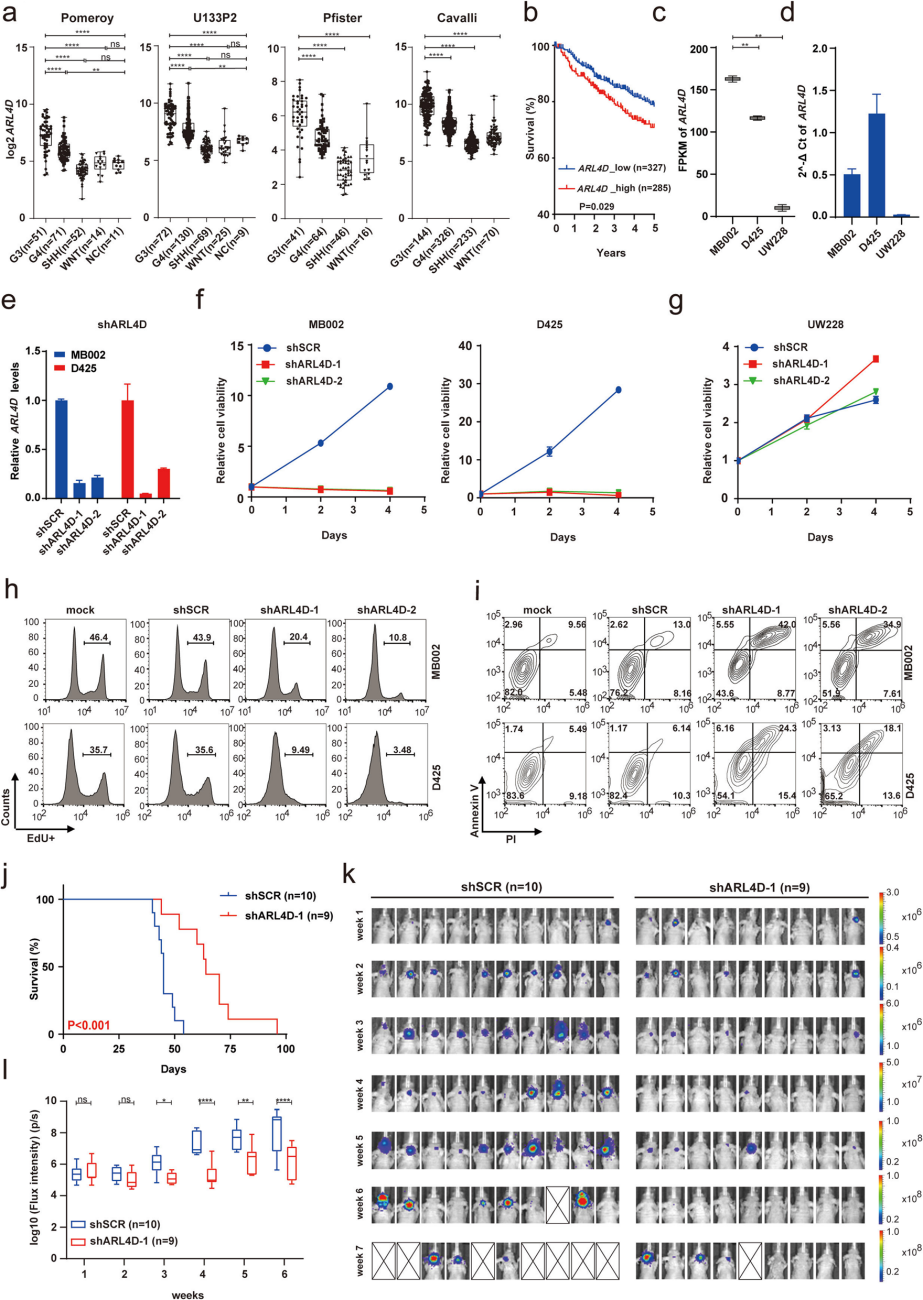
Verification of *ARL4D* as a subtype-specific tumor dependency of G3-MB. **a** Box plots showing *ARL4D* mRNA levels of four MB subgroups or NC in the indicated MB datasets. **b** Kaplan–Meier analysis of the overall survival of MB patients stratified by *ARL4D* expression level in Cavalli dataset. **c** Box plots showing the FPKM values of *ARL4D* from two RNA-Seq replicates of MB002, D425 lines versus UW228. **d** RT-qPCR analysis of *ARL4D* in MB002, D425 and UW228 lines. MB002 and D425 cells upon *ARL4D* knockdown were subjected to RT-qPCR analysis (**e**) and cell viability measurement at Day 0/2/4 (**f**). **g** UW228 cells were infected with shSCR or two separate clones of shARL4D lentiviruses individually at the same MOI as cells in **e-f**. Day 0-normalized cell viability was measured at Day 0/2/4. **h**-**i** FACS analyses of cell proliferation (**h**) and apoptosis (**i**) of MB002 and D425 cells upon *ARL4D* knockdown. **j** Kaplan-Meier survival curve of nude mice carrying orthotopic xenografts of MB002-GFP-luc cells stably expressing shARL4D-1 or shSCR. **k**-**l** Tumor growth of xenografted nude mice treated as described in (**j**) was monitored by IVIS weekly. The collected mice images with corresponding signal scale bars measured in p/s are shown in (**k**). The crossed white box indicates death of the treated mouse. Box plots showing the signals of total bioluminescence flux intensity for xenografted nude mice of each treatment condition and the data are presented as mean ± SEM in (**l**). All RT-qPCR and cell viability assays were performed in triplicate and the data are presented as mean ± SD. Statistical significance was determined by one-way ANOVA (**a**), two-sided log-rank test (**b** and **j**), two-tailed *t* test (**c**) and two-way ANOVA (**l**)

To demonstrate the proof of principle that novel therapeutic targets could be unveiled from the identified SE-driven core transcriptional regulatory network, *ARL4D*, one of the eleven newly identified downstream effector vSE genes, was selected for further investigation. ARL4D is a member of the ADP-ribosylation factor (ARF) family of proteins that belongs to the RAS superfamily of small GTPases. ARF family members, which usually play functions in cytoskeleton remodeling, cell cycle, cell migration and adhesion in normal tissues, are frequently found to be subverted by cancer for regulating proliferation, migration and invasion of tumor cells [44]. Even though ARL4D was previously identified as a glioma-associated antigen dependent on loss of PTEN and consequent activation of Akt/mTOR pathway [45, 46], its oncogenic roles and underlying molecular mechanisms have never been reported in any cancer type before. As shown in Fig. 5a-b, *ARL4D* is consistently and significantly upregulated in G3-MB versus NC or the other MB subtypes and patients with higher *ARL4D* levels exhibit significantly worse prognosis. Single-cell transcriptomic analysis also showed that G3-MB tumor cells exhibited much stronger *ARL4D* expression than tumor cells of the other three MB subtypes (Fig.S6a). Moreover, GP3-C2, the photoreceptor differentiated tumor cell cluster of G3-MB, exhibits the highest expression of *ARL4D* among all the identified tumor cell clusters (Fig.S6a). To be noted, *CRX* and *OTX2*, the potential upstream SE-associated TFs of *ARL4D* described in Fig. 2h, were found to be enriched in GP3-C2 as well (Fig.S6a). We then compared the expression and tumor-dependency of *ARL4D* in D425 and MB002 versus UW228. Our results showed the two G3-MB lines expressed much higher level of *ARL4D* than UW228 (Fig. 5c-d), and knockdown of *ARL4D* with shRNAs or cas13d-sgRNAs only markedly suppressed growth of D425 and MB002 but not UW228 cells in vitro (Fig. 5e-g and S7a-c). *ARL4D* loss induced growth inhibition of G3-MB cells resulted from disruption of proliferation and induction of apoptosis of tumor cells (Fig. 5h-i and S7d-e). Furthermore, we showed knockdown of *ARL4D* caused marked growth disruption of MB002-GFP-luc xenograft model in vivo and significantly prolonged the survival of xenografted mice (Fig. 5j-l). Taken together, our results verified *ARL4D* as a subtype-specific tumor-dependency of G3-MB.

**Fig. 6:**
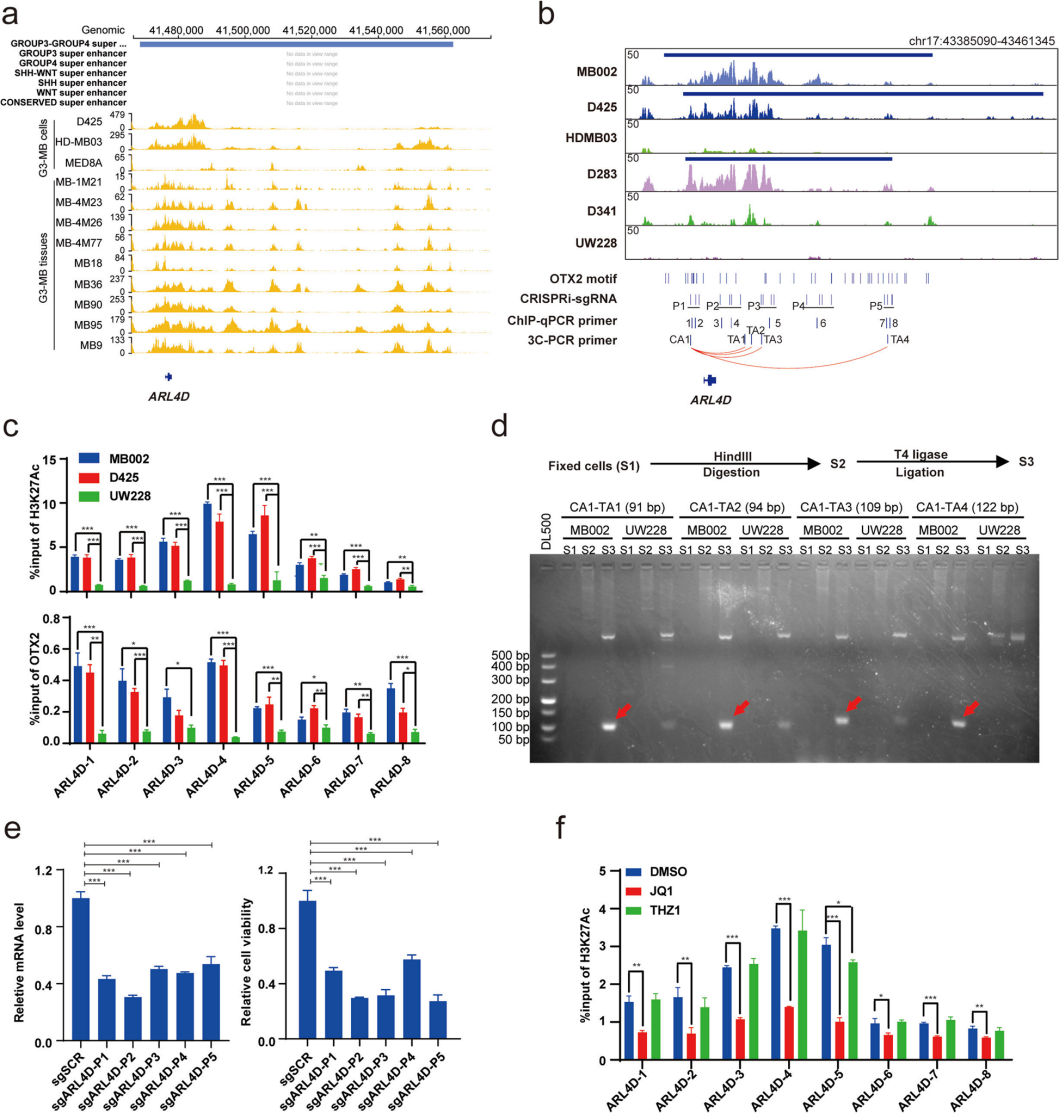
Transcriptional regulation of ARL4D by subtype-specific SE and SE-associated TF in G3-MB. **a** Gene tracks of H3K27Ac ChIP-seq signal across G3-MB cells and tissues at proximal SE regions of *ARL4D*. SE region is depicted in colored line over the gene tracks. The gene track images were obtained from https://viz.stjude.cloud/st-jude-childrens-research-hospital/visualization/chip-seq-landscape-of-primary-medulloblastomas~23. **b** Gene tracks of H3K27Ac ChIP-seq signal across five G3-MB lines and non-G3-MB line UW228 at proximal SE regions of *ARL4D*. SE regions are depicted in colored lines over the gene tracks. The positions of predicated binding motifs of OTX2, ChIP-qPCR amplicons, pooled-CRISPRi sgRNA targeting regions and 3C-PCR primers are shown below the tracks. **c** ChIP-qPCR analysis of H3K27Ac (top) and OTX2 (bottom) enrichment near *ARL4D* in MB002, D425 and UW228. Tested positions of amplicons are shown in (**a**). **d** 3C-PCR analysis of chromatin interaction between the H3K27Ac peak regions within SE and promoter of *ARL4D* in MB002 and UW228. The workflow of 3C-PCR was shown above the agarose gel electrophoresis of 3C-PCR products. Positions of 3C-PCR primers are shown in **a**. **e** Pooled-CRISPRi sgRNAs are designed to target the SE regions of *ARL4D* in stable dCas9-KRAB expressing MB002, the *ARL4D* expression level (left) is measured by RT-qPCR, and cell viabilities (right) are measured at Day 6. The pooled-CRISPRi sgRNA targeting regions are shown in (**a**). **f** ChIP-qPCR analysis of H3K27Ac enrichment near *ARL4D* in MB002 treated with 2 μM JQ1 or 0.05 μM THZ1 for 24 h, respectively. All cell viability, ChIP-qPCR and RT-qPCR assays were performed in triplicate and the data are presented as mean ± SD. Statistical significance was determined by one-way ANOVA (**c** and **f**) and two-tailed *t* test (**e**)

To explore the transcriptional regulation of *ARL4D* in G3-MB, we firstly examined the H3K27Ac ChIP-seq signals around *ARL4D* genomic locus across multiple G3-MB tissues and cell lines. UW228 was analyzed in parallel as control. As shown in Fig. 6a-b, *ARL4D* represents a G3/G4-MB SE at tumor tissue level and exhibits robustly elevated H3K27Ac signals in G3-MB lines versus UW228. As a result, it is identified as a SE-associated target gene in D425, MB002 and D283 lines or top-ranked TE-associated target gene in HD-MB03 and D341 lines (Fig.S8a). In contrast, *ARL4D* only ranked 45.9% from the top within all the TE-associated target genes in UW228 line (Fig.S8a). After obtaining the commercially-available ChIP-qualified anti-OTX2 antibody reported in a previous study [29], we performed ChIP-qPCR analyses to confirm the enrichment of H3K27Ac and OTX2 at *ARL4D*’s SE regions in D425 and MB002 cells versus UW228 cells (Fig. 6b-c). Then we performed 3C-PCR analysis with two different restriction enzyme digestion, HindIII (Fig. 6b, d and S8b) and MboI (Fig.S8c-e), to demonstrate the chromatin looping between *ARL4D*’s SE and promoter regions in G3-MB cells. We also performed CRISPRi analysis with pooled sgRNAs targeting *ARL4D*’s SE regions and the results showed CRISPRi silencing of *ARL4D*’s SE could significantly impair its transcription and the growth of G3-MB cells (Fig. 6e). When ChIP-qPCR analysis with anti-H3K27Ac antibody was performed on JQ1 or THZ1 treated MB002 cells to measure their impact on *ARL4D*’s SE, we found JQ1 but not THZ1 could significantly reduce the enrichment of H3K27Ac signal at *ARL4D*’s SE regions, supporting the direct targeting of SE by BET inhibition (Fig. 6f). Furthermore, we measured the impact of *OTX2* knockdown on the enrichment of H3K27Ac and OTX2 at *ARL4D*’s SE regions in MB002 cells. As shown in Fig.S8f, while the binding of OTX2 was broadly abrogated, the H3K27Ac enrichment was partially impaired in only one of the tested regions, suggesting OTX2 might play a dominant role in this region of *ARL4D*’s SE (Fig. 6f).

**Fig. 7:**
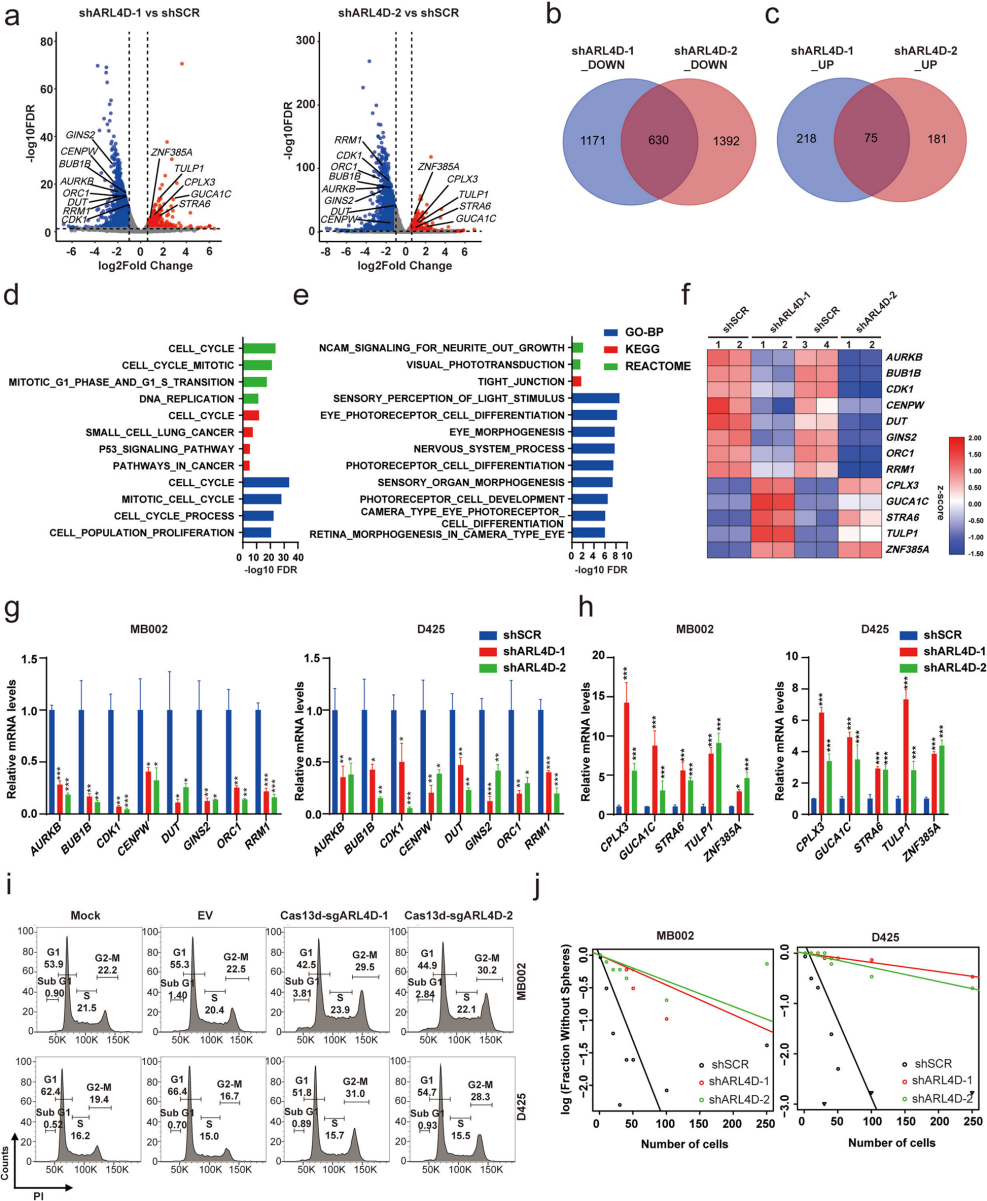
*ARL4D* is required for maintaining cell cycle progression and inhibiting neural differentiation of G3-MB cells. **a** Volcano plots showing significantly altered genes (mean FPKM of shSCR or shARL4D ≥ 1, log2_FC < -1 or > 0.6, FDR < 0.05) in MB002 cells upon *ARL4D* knockdown by two separate shRNA clones. Selected cell cycle and neural development related genes for further validation are shown. **b**-**c** Venn diagram analysis of significantly downregulated (**b**, mean FPKM of shSCR ≥ 1, log2_FC < -1, FDR < 0.05) or upregulated genes (**c**, mean FPKM of shARL4D ≥ 1, log2_FC > 0.6, FDR < 0.05) in MB002 cells upon *ARL4D* knockdown by two separate shRNA clones. **d-e** GO (BP, biological processes) and Pathway (KEGG and REACTOME) analyses of the shared downregulated (**d**) or upregulated genes (**e**) identified in (**b**) and (**c**), respectively. **f** Heatmap of gene expression levels of the selected cell cycle and neural development related genes that are significantly downregulated or upregulated upon *ARL4D* knockdown in MB002 cells by two separate shRNA clones. **g**-**h** RT-qPCR validation of the selected significantly differentially expressed cell cycle (**g**) or neural development (**h**) related genes tested in (**f**) upon *ARL4D* knockdown by two separate shRNA clones in MB002 and D425 cells, respectively. **i** FACS analysis of cell cycle of MB002 and D425 cells with *ARL4D* being knocked down following infection of two separate clones of Cas13d-sgARL4D lentivirus. Tumor cells stably expressing Cas13d empty vector (EV) and uninfected tumor cells (Mock) were analyzed in parallel as control. **j** Limiting dilution analysis of the frequency of tumorsphere forming cells of MB002 and D425 cells following *ARL4D* knockdown by two separate shRNA clones. All RT-qPCR assays were performed in triplicate and the data are presented as mean ± SD. Statistical significance was determined by one-way ANOVA (**g**-**h**)

To dissect the molecular mechanism underlying *ARL4D*’s tumor-dependency of G3-MB, we performed RNA-seq analysis of MB002 cells stably expressing two separate shARL4D clones or scramble control shRNA (Fig. 7a). The 630 commonly downregulated genes (log2FC < -1, FDR < 0.05) shared by the two shARL4D clones were found to be enriched in cell cycle related biological processes whereas the 75 commonly upregulated genes (log2FC > 0.6, FDR < 0.05) were enriched in neural cell differentiation and development related biological processes (Fig. 7b-e). We then performed RT-qPCR verification of eight commonly downregulated cell cycle-related genes (*AURKB*, *BUB1B*, *CDK1*, *CENPW*, *DUT*, *GINS2*, *ORC1*, *RRM1*) and five commonly upregulated nervous system development-related genes (*CPLX3*, *GUCA1C*, *STRA6*, *TULP1*, *ZNF385A*) selected based on the RNA-seq data in MB002 and D425 cells upon knockdown of *ARL4D* (Fig. 7f-h). Furthermore, we showed loss of *ARL4D* caused cell cycle arrest at G2/M phase and significantly attenuated tumor-sphere formation in both D425 and MB002 lines (Fig. 7i-j and S8g-h). Collectively, our results demonstrated that *ARL4D*, which is required for maintaining cell cycle progression and inhibiting neural differentiation of tumor cells, represents a novel SE-associated subtype-specific tumor-dependency and therapeutic target of G3-MB.

## Discussion:

In this study, we chose to deeply dissect SE-driven transcriptional dependencies of G3-MB to better understand its tumor biology and identify novel SE-associated therapeutic strategies or targets. Even though it has been reported before there are poor overlap and correlation between enhancer landscapes of primary tumor tissues and patient-derived tumor cell lines of MB [12], here we were able to show the conserved SE-associated oncogenic signature between primary tumor lines and tissues of G3-MB was enriched of subtype-specific upregulated tumor-dependent genes and MB patients harboring enrichment of those transcripts exhibited worse prognosis. We then built G3-MB’s SE-driven core transcriptional regulatory network composed of fourteen such conserved SE-associated subtype-specific upregulated tumor-dependent genes, including three well-recognized TFs (*MYC*, *OTX2*, *CRX*) and eleven newly identified downstream effector genes (*ARL4D*, *AUTS2*, *BMF*, *IGF2BP3*, *KIF21B*, *KLHL29*, *LRP8*, *MARS1*, *PSMB5*, *SDK2* and *SSBP3*). Moreover, we revealed BETi and CDK7i, which were previously reported to effectively suppress G3-MB [14–17], both exhibited anti-SE activity against G3-MB cells as they did in many other cancer types [7]. These results verified the oncogenic role of SE-driven transcriptional dependencies in G3-MB and supported us to further explore its therapeutic potential by searching for other SE-associated therapeutic strategies or targets.

There have been multiple effective anti-SE therapeutic strategies reported in various cancer types via targeting SE complex components and SE-associated effector genes individually or in combination [8–11]. We noticed that only *PSMB5* within our identified SE-driven core transcriptional regulatory network of G3-MB has targeted small-molecule inhibitor and *PSMB5*-targeted PSI drug Marizomib has been reported to effectively inhibit growth of G3/G4-MB alone or in combination with radiation in vitro [39]. Therefore, we evaluated the therapeutic effects with pairwise combinations of THZ1, JQ1 and Marizomib on treating multiple G3-MB lines and synergy was detected between JQ1 with THZ1 or Marizomib but not THZ1 with Marizomib. Mechanistically, we revealed that the combinations of BETi with CDK7i or PSI exerted their synergistic inhibitory effects via stronger suppression of SE-associated transcription or higher activation of ER stress and unfolded protein response (UPR), respectively, sharing very similar molecular mechanisms with previously reported cancer types [8, 9, 42, 43]. Notably, PSI, CDK7i and BETi drugs have all entered human clinical trials for cancer therapy. More importantly, PSI drug Marizomib and BETi drug OTX015 have been shown to possess sufficient brain penetration capacity [40, 41, 47]. Therefore, our identified combinatory anti-SE strategies exhibit great potential for future clinical application.

It has been proven that novel therapeutic targets can be unveiled from SE-associated downstream effector genes [10, 11]. Accordingly, *ARL4D*, a member of the newly identified SE-driven core transcriptional regulatory network of G3-MB with very little prior knowledge in cancer, was subjected to further investigation. Notably, small GTPase family members used to be considered as undruggable, but plenty of new approaches or strategies have been developed in recent years for targeting GTPase proteins directly or indirectly via their modulators [44, 48], thus making ARL4D a plausible therapeutic target for future drug development. As a result, an OTX2-SE-*ARL4D* regulatory axis is revealed to represent an important subtype-specific tumor dependency of G3-MB via contributing to maintaining cell cycle progression and repressing neural differentiation. As a oncogenic driver TF of G3-MB [49], OTX2 has been previously shown to promote tumor cell cycle progression via direct activation of multiple cell cycle genes and inhibit neural differentiation via repressing transcription of various neurodevelopmental genes directly or indirectly [50–53]. Hence, our results illustrate ARL4D as another crucial downstream oncogenic effector of OTX2. On the other hand, CRX was also found to be a potential upstream TF of ARL4D in G3-MB (Fig. 2g-h). Even though it could not be experimental verified due to the lack of commercially available ChIP-qualified CRX antibody, our data are in line with a previous study that reports the oncogenic role of NRL and CRX in subtype-specific aberrant activation of photoreceptor differentiation program [13]. In that study, *ARL4D* is identified to be one of the 385 high confidence SE-associated genes containing NRL and CRX motifs in proximity and its transcript level is significantly downregulated in NRL knockdown D458 cells. Moreover, our scRNA-seq data analysis also revealed *ARL4D*, *OTX2* and *CRX* were all enriched in GP3-C2, the photoreceptor differentiated tumor cell cluster of G3-MB defined in a recent single-cell transcriptomic study of MB [38]. Intriguingly, we noticed that the top significantly upregulated transcriptome signatures upon *ARL4D* knockdown in G3-MB cells were mostly related to photoreceptor differentiation as well (Fig. 7e), suggesting *ARL4D* might be required for restraining the aberrant activation of photoreceptor differentiation program at a proper level. To be noted, *ARL4D* is also significantly upregulated in G4-MB versus normal cerebellum (Fig. 5a). Like GP3-C2, GP4-C2, the photoreceptor differentiated tumor cell cluster of G4-MB, exhibits the highest expression of *ARL4D* and highly expresses *OTX2* and *CRX* (Fig.S6a). Therefore, it would be interesting to test in future whether *ARL4D* also works as an essential gene and is transcriptionally regulated by OTX2 and CRX as well in *ARL4D*-high G4-MB tumors if proper tumor models are available.

## Conclusion

In summary, this study utilizes the conserved SE-associated tumor-dependent gene signatures between primary tumor tissues and patient-derived tumor cell lines to dissect the oncogenic role and therapeutic potential of SE-driven transcriptional dependencies of G3-MB, resulting in better understanding of its tumor biology and identification of novel therapeutic strategies and targets. To be noted, other than *ARL4D* and *PSMB5*, the other newly identified SE-associated tumor-dependent effector genes of G3-MB are worthy of further investigation as well. For instance, the oncofetal RNA-binding protein IGF2BP3 was recently identified as a m6A reader [54]. The roles and related mechanisms of RNA epigenetic modifications like m6A in G3-MB remains unclear and deserves further investigation.

## Supplementary Information

**Additional file 1: Supplementary Table S1**

**Additional file 2: Supplementary Table S2**

**Additional file 3: Supplementary Table S3**

**Additional file 4: Supplementary Table S4**

**Additional file 5: Supplementary Table S5**

**Additional file 6: Supplementary Table S6**

**Additional file 7: Fig. S1.** **a**-**b** Violin plots showing GSVA score of cSE (**a**) or tSE (**b**) signature genes in four MB subgroups or NC of the indicated MB datasets. (**c**-**d**) Kaplan-Meier survival analysis of the GSVA scores of cSE (**c**) or tSE (**d**) genes in Cavalli dataset of MB. The patient cohort was stratified as high versus low groups by median GSVA score. Statistical significance was determined by one-way ANOVA (**a**-**b**), two-sided log-rank test (**c**-**d**). **Fig. S2.** **a**-**c** RT-qPCR analysis of vSE genes in MB002 or D425 cells when* MYC* (**a**), *OTX2* (**b**) and *CRX* (**c**) were knocked down by shRNA individually. shSCR served as control. RT-qPCR assays were performed in triplicate and the data are presented as mean ± SD. Statistical significance was determined by two-tailed unpaired *t* test (**a**-**c**). **Fig. S3.** **a** Gene tracks of H3K27Ac ChIP-seq signal across G3-MB cells and tissues at SE regions near* PSMB5*. SE region is lined out over the gene tracks. **b** Gene tracks of H3K27Ac ChIP-seq signal across five G3-MB lines and non-G3-MB cells UW228 at SE regions near* PSMB5*. SE regions are lined out over the gene tracks. Positions of tested ChIP-qPCR amplicons, pooled-CRISPRi sgRNA targeting regions and 3C-PCR primers are shown below the tracks. **c** Box plots showing the FPKM values of *PSMB5* from RNA-Seq replicates of D283, D425 and MB002 lines versus UW228, a control non-G3-MB line. **d** ChIP-qPCR analysis of H3K27Ac enrichment near *PSMB5* in MB002, D283 and UW228, respectively. **e** 3C-PCR analysis of chromatin interaction between the H3K27Ac peak regions and promoter of *PSMB5* in MB002, D283 and UW228. The workflow of 3C-PCR was shown in top panels, the agarose gel electrophoresis of 3C-PCR products was shown in bottom panels. The positions of 3C-PCR primers are shown in (**b**). **f** Negative control SE region of PSMB5 (NCP) without MboI site of 3C-PCR analysis related to Fig. S3 (e) was tested in MB002, D283 and UW228 by RT-qPCR. **g**-**h** Pooled-CRISPRi sgRNAs are designed to target the SE regions of* PSMB5 *in stable dCas9-KRAB expressing MB002 and D283, the *PSMB5 *expression level (**g**) is measured by RT-qPCR, and cell viabilities (**h**) are measured at Day 6. **i** RT-qPCR analysis of *MYC* and *PSMB5* in D283 cells treated with 1 μM JQ1 or 0.1 μM THZ1 for 6 h. RT-qPCR analysis of *PSMB5* in D283 when* MYC *(**j**), *OTX2* (**k**) and *CRX* (**l**) were knocked down by shRNA individually (left). The cell viabilities were also measured (right). All RT-qPCR and cell viability assays were performed in triplicate and the data are presented as mean ± SD. Statistical significance was determined by one-way ANOVA (**d**) and two-tailed *t* test (**c**) and (**g**-**i**). **Fig. S4.** **a** UMAP plot showing MB subtypes (top left) or expression levels of *PSMB5* in MB (top right) and GP3-MB (middle left),* MYC* (middle right), *OTX2 *(bottom left) and *CRX *(bottom right) in GP3-MB.** Fig. S5**. **a** Cell viability (top) and CI (bottom) of MB002 and D425 cells treated with THZ1 and Marizomib individually or in combination at the indicated concentrations for 72 h. **b** Cell viability (top) and CI (bottom) of D425 cells treated with JQ1 and Marizomib individually or in combination at the indicated concentrations for 72 h (top). **c** Cell viability of MB002 and D425 cells treated with Marizomib (3.25 nM for MB002, 125 nM for D425) and JQ1 (500 nM) individually or in combination were measured at Day 0/2/4 post treatment and normalized to Day 0-value. **d** The body weight of xenograft nude mice described in Fig. 4 (**f**-**h**) was recorded. Cell viability assays were performed in triplicate and the data are presented as mean ± SD. Line plot showing body weight of xenograft nude mice was presented as mean ± SEM in (**d**). **Fig. S6.** **a** UMAP plot showing MB subtypes (top left) or expression levels of *ARL4D* in MB (top right), GP3-MB (middle left) and GP4-MB (middle right),* OTX2 *(bottom two rows, left) and *CRX *(bottom two rows, right) in GP3- or GP4-MB. **Fig. S7.** **a**-**b** MB002 and D425 cells were infected with Cas13d empty vector (EV) or two separate clones of Cas13d-sgARL4D lentiviruses individually. After puromycin selection, the infected cells were then subjected to RT-qPCR analysis of *ARL4D* (**a**) as well as cell viability measurement at Day 0/2/4 (**b**). Uninfected tumor cells (Mock) were analyzed in parallel. RT-qPCR and cell viability results were normalized to EV or Day 0 sample, respectively. **c** UW228 cells were infected with Cas13d empty vector (EV) or two separate clones of Cas13d-sgARL4D lentivirus individually at the same MOI as MB002 and D425 in (**a**-**b**). Cell viability were measured at Day 0/2/4 and normalized to Day 0 sample. **d**-**e** FACS analyses of cell proliferation (**d**) and apoptosis (**e**) of MB002 and D425 cells upon *ARL4D* knockdown with Cas13d-sgARL4D. All RT-qPCR and cell viability assays were performed in triplicate and the data are presented as mean ± SD. **Fig. S8.** **a** Percentage ranking analysis of *ARL4D* as SE- or TE-target gene in five G3-MB cells and non-G3-MB cells UW228 was shown in the table. **b** Negative control SE region of *ARL4D* (NCA) without HindIII site of 3C-PCR analysis related to Fig. 6 (**d**) was tested in MB002 and UW228 by RT-qPCR. **c**-**e** 3C-PCR analysis of chromatin interaction between the H3K27Ac peak regions and promoter of the *ARL4D* in MB002. The positions of 3C-PCR primers are shown in (**c**). The same NCA region as above (neither containing the MboI site) was tested by RT-qPCR (**d**). The workflow of 3C-PCR was shown over the electrophoretogram of 3C-PCR products (**e**). **f** ChIP-qPCR analysis of OTX2 (top) and H3K27Ac (bottom) enrichment near* ARL4D* in MB002 upon OTX2 knockdown. **g** Phase changes of cell cycle in MB002 and D425 following *ARL4D* knockdown with two separate Cas13d-sgARL4D clones were determined by FACS as described in Fig. 7 (**i**). **h** The frequencies of tumorsphere-forming cells of MB002 and D425 upon *ARL4D* knockdown with two separate shRNA clones were determined by limiting dilution assay as described in Fig.7 (**j**) and analyzed by L-Calc^TM^ software. ChIP-qPCR assay was performed in triplicate and the data are presented as mean ± SD. Statistical significance was determined by one-way ANOVA (**f**).

## Data Availability

The ChIP-seq and RNA-seq data discussed in this publication have been deposited in NCBI’s Gene Expression Omnibus and are accessible through GEO series accession number GSE185025.

## Abbreviations

ARF: ADP-ribosylation factor
BBB: Blood–brain barrier
BETi: BET inhibitor
CDK7i: CDK7 inhibitor
ChIP: Chromatin immunoprecipitation
CRISPRi: CRISPR interference
cSE: Cellular_SE-associated_gene_signature
ELDA: Extreme limiting dilution assay
G3-MB: Group 3 subtype medulloblastoma
GSEA: Gene set enrichment analysis
GSVA: Gene set variation analysis
IVIS: In vivo imaging system
MB: Medulloblastoma
NC: Normal cerebellum
oSE: Overlapping_SE-associated_gene_signature
PSI: Proteasome inhibitor
ROSE: Rank-ordering super-enhancers
SE: Super-enhancer
TE: Typical enhancer
TF: Transcription factor
tSE: Tissue_SE-associated_gene_signature
UPR: Unfolder protein response
vSE: Vital_SE-associated_gene_signature
3C-PCR: Chromosome conformation capture coupled with PCR
