# Supplementary material for: Dissecting super-enhancer driven transcriptional dependencies reveals novel therapeutic strategies and targets for group 3 subtype medulloblastoma
Source: J Exp Clin Cancer Res. 2022 Oct 22;41:311. doi: 10.1186/s13046-022-02506-y (PMC9587669; doi:10.1186/s13046-022-02506-y)
Supplement: Supplementary file 7 — Additional file 7: Fig. S1. a-b Violin plots showing GSVA score of cSE (a) or tSE (b) signature genes in four MB subgroups or NC of the indicated MB datasets. (c-d) Kaplan-Meier survival analysis of the GSVA scores of cSE (c) or tSE (d) genes in Cavalli dataset of MB. The patient cohort was stratified as high versus low groups by median GSVA score. Statistical significance was determined by one-way ANOVA (a-b), two-sided log-rank test (c-d). Fig. S2. a-c RT-qPCR analysis of vSE genes in MB002 or D425 cells when MYC (a), OTX2 (b) and CRX (c) were knocked down by shRNA individually. shSCR served as control. RT-qPCR assays were performed in triplicate and the data are presented as mean ± SD. Statistical significance was determined by two-tailed unpaired t test (a-c). Fig. S3. a Gene tracks of H3K27Ac ChIP-seq signal across G3-MB cells and tissues at SE regions near PSMB5. SE region is lined out over the gene tracks. b Gene tracks of H3K27Ac ChIP-seq signal across five G3-MB lines and non-G3-MB cells UW228 at SE regions near PSMB5. SE regions are lined out over the gene tracks. Positions of tested ChIP-qPCR amplicons, pooled-CRISPRi sgRNA targeting regions and 3C-PCR primers are shown below the tracks. c Box plots showing the FPKM values of PSMB5 from RNA-Seq replicates of D283, D425 and MB002 lines versus UW228, a control non-G3-MB line. d ChIP-qPCR analysis of H3K27Ac enrichment near PSMB5 in MB002, D283 and UW228, respectively. e 3C-PCR analysis of chromatin interaction between the H3K27Ac peak regions and promoter of PSMB5 in MB002, D283 and UW228. The workflow of 3C-PCR was shown in top panels, the agarose gel electrophoresis of 3C-PCR products was shown in bottom panels. The positions of 3C-PCR primers are shown in (b). f Negative control SE region of PSMB5 (NCP) without MboI site of 3C-PCR analysis related to Fig. S3 (e) was tested in MB002, D283 and UW228 by RT-qPCR. g-h Pooled-CRISPRi sgRNAs are designed to target the SE regions of PSMB5 in stable dCas9-KRAB ex [file 13046_2022_2506_MOESM7_ESM.pdf]

Li_et_al_Supplementary_Fig.S1

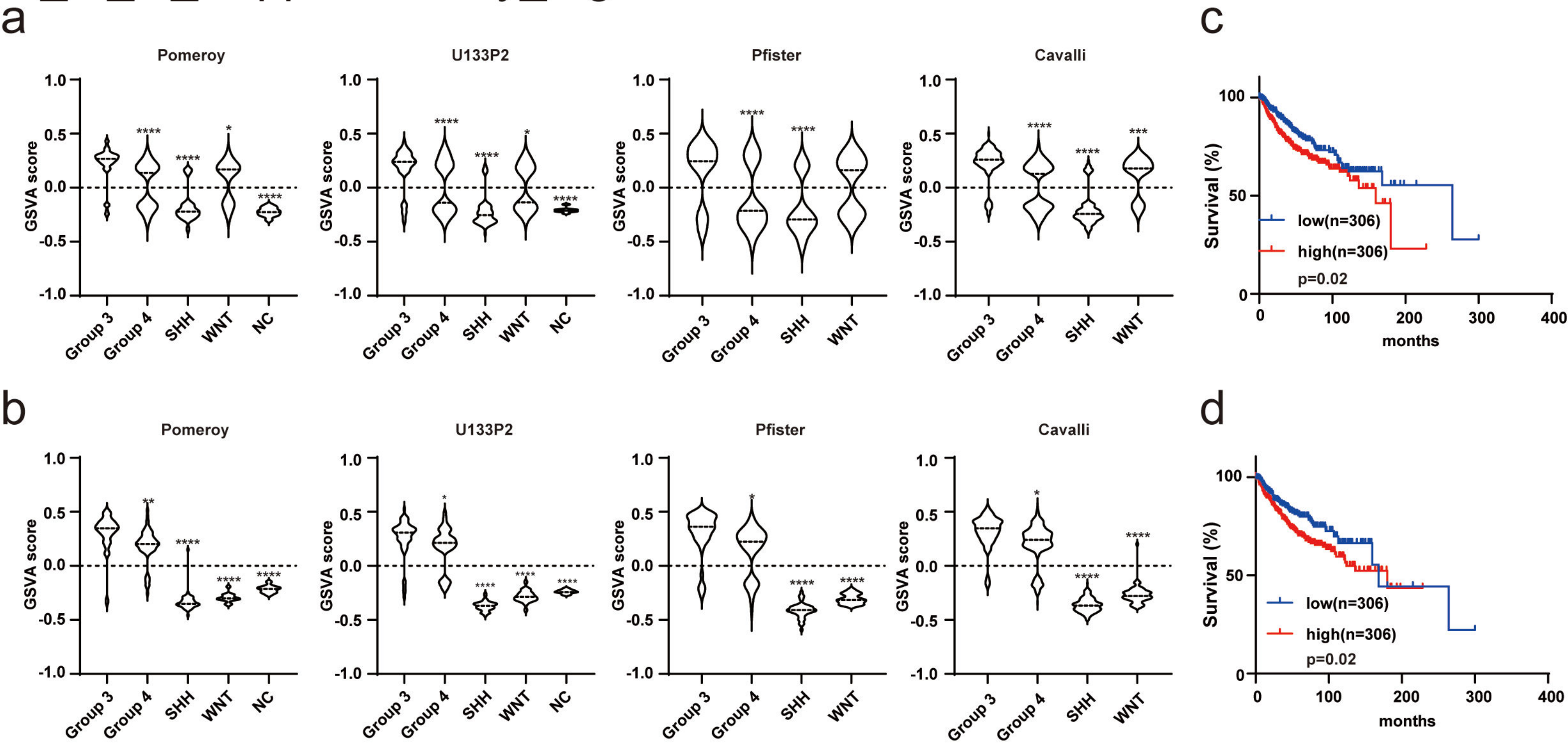

Li_et_al_Supplementary_Fig.S2

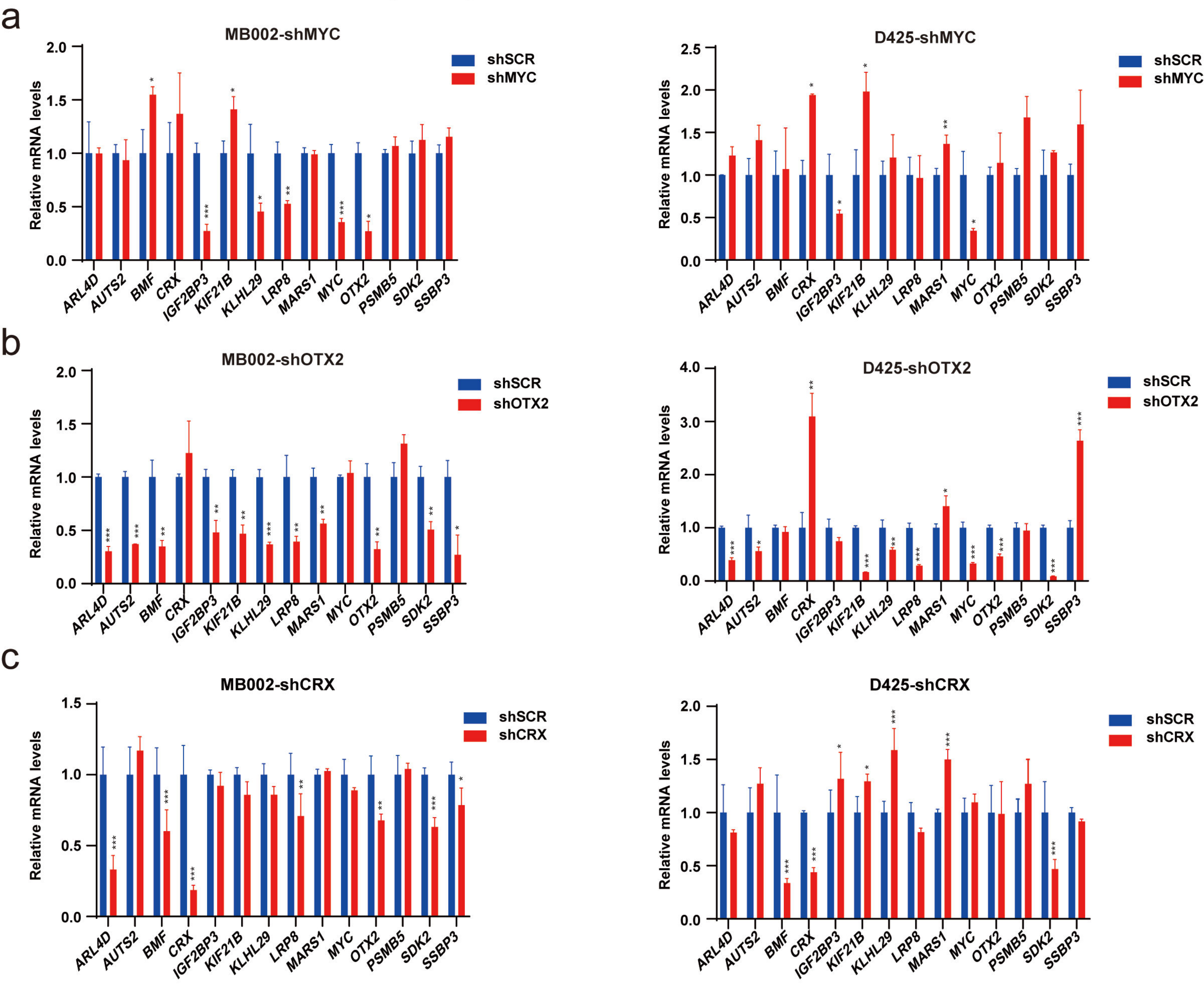

Li_et_al_Supplementary_Fig.S3

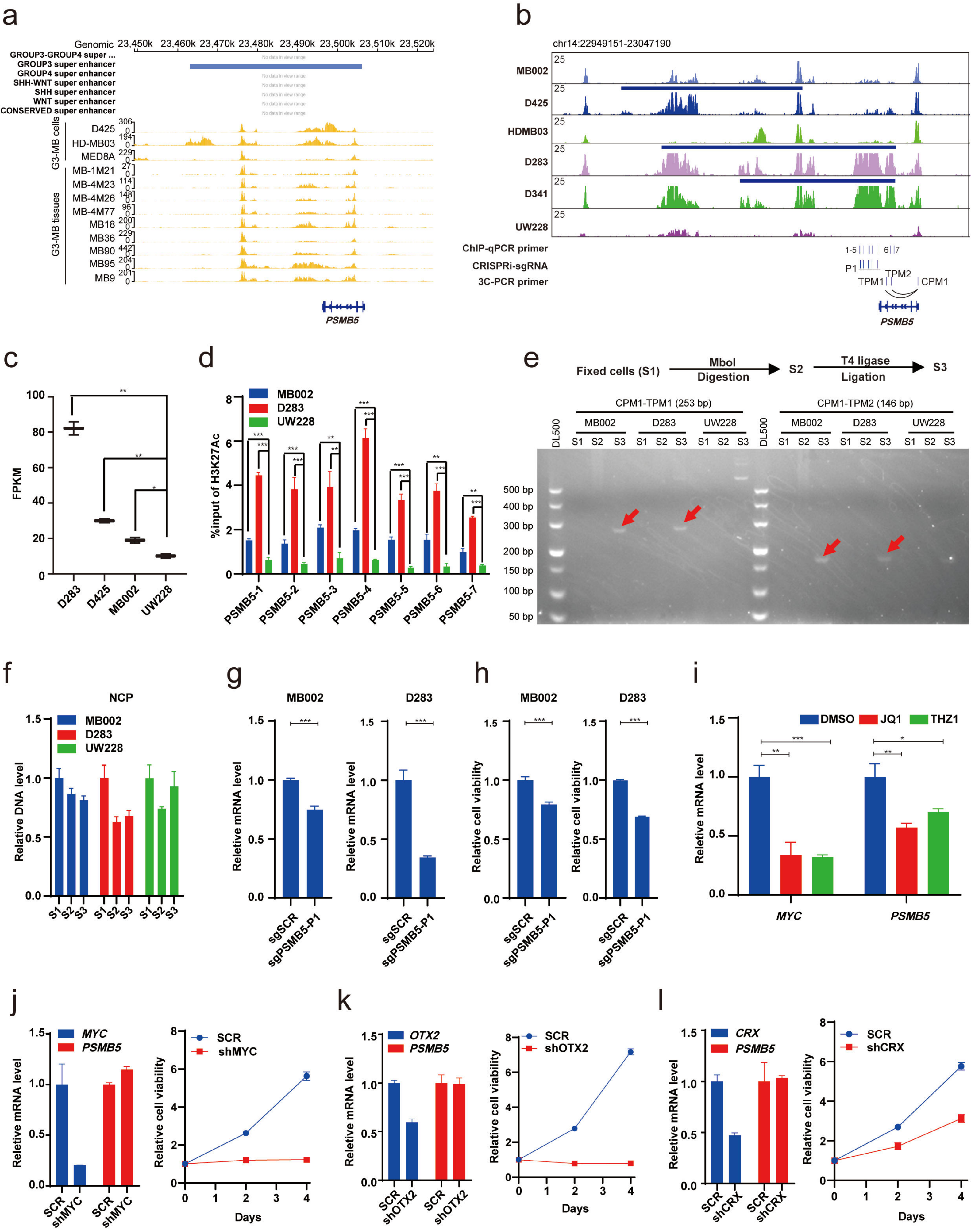

Li_et_al_Supplementary_Fig.S4

a

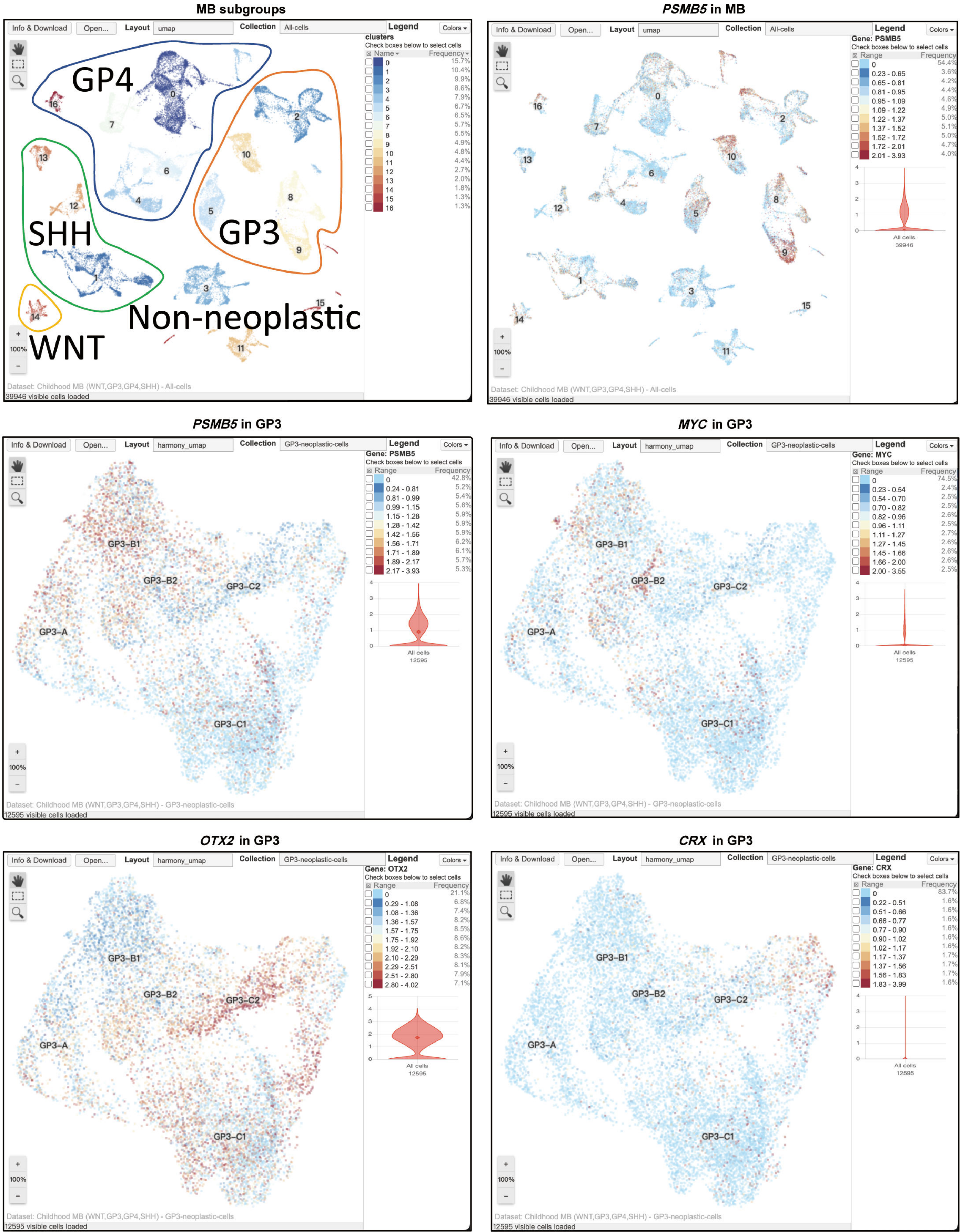

Li_et_al_Supplementary_Fig.S5

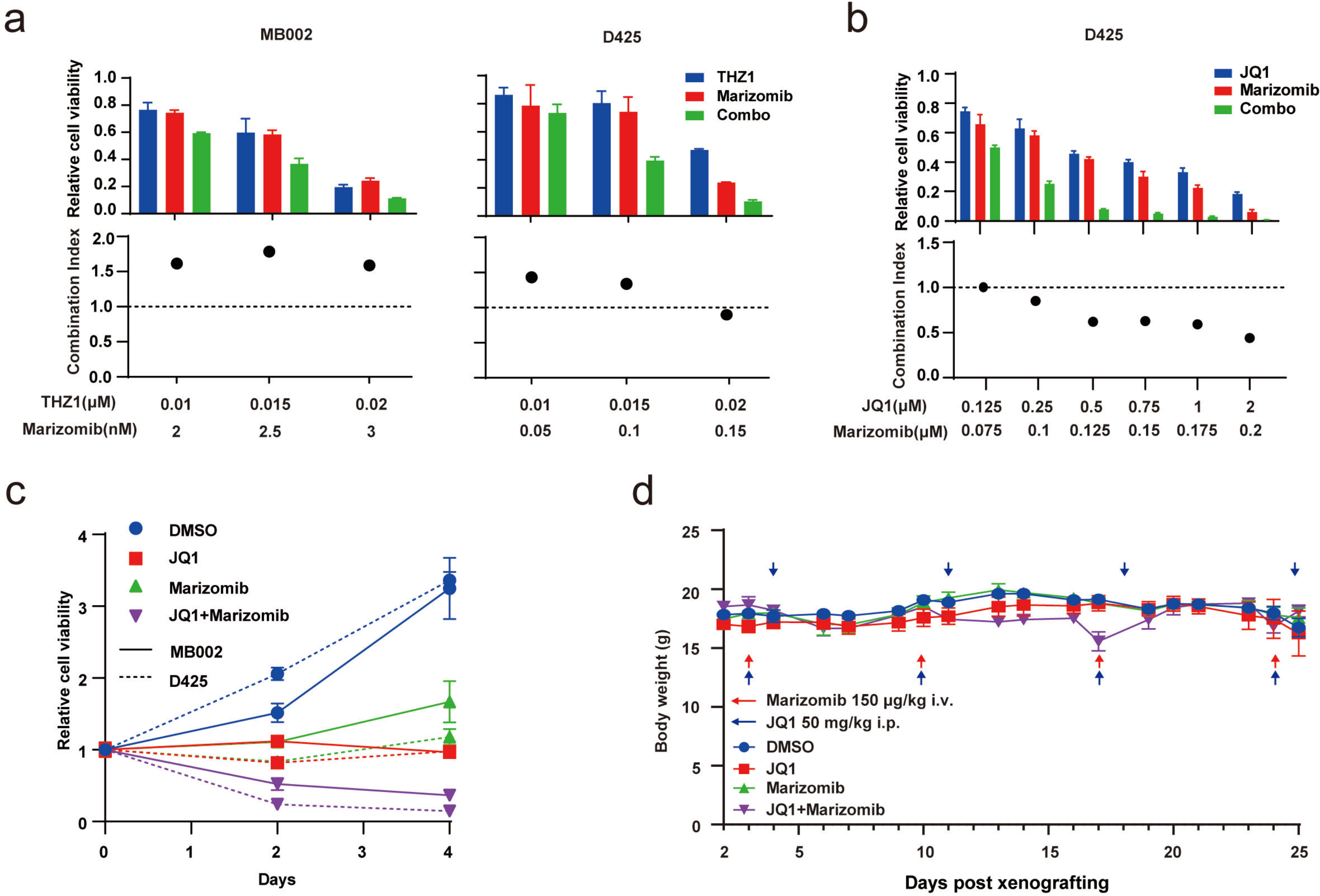

Li_et_al_Supplementary_Fig.S6

a

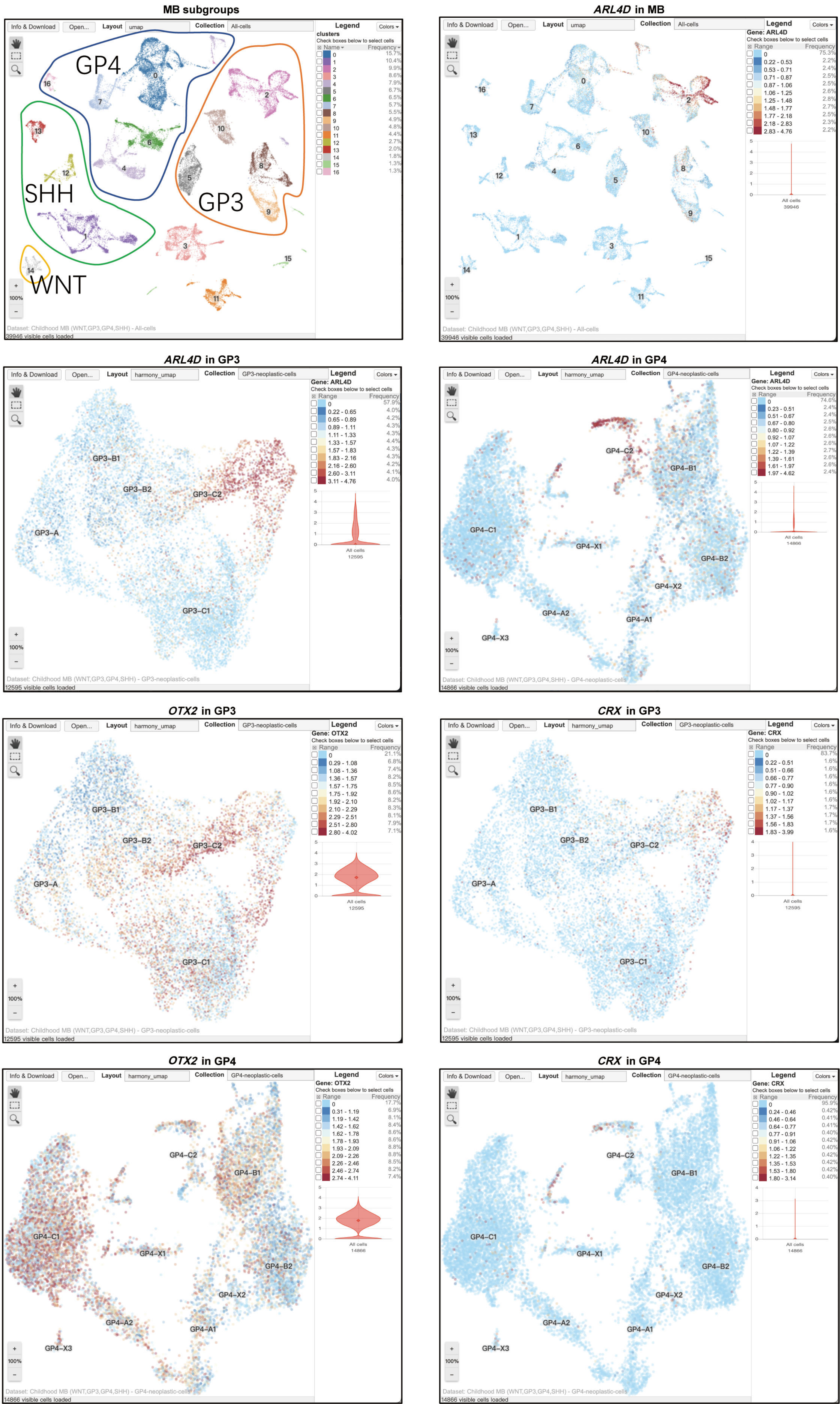

Li_et_al_Supplementary_Fig.S7

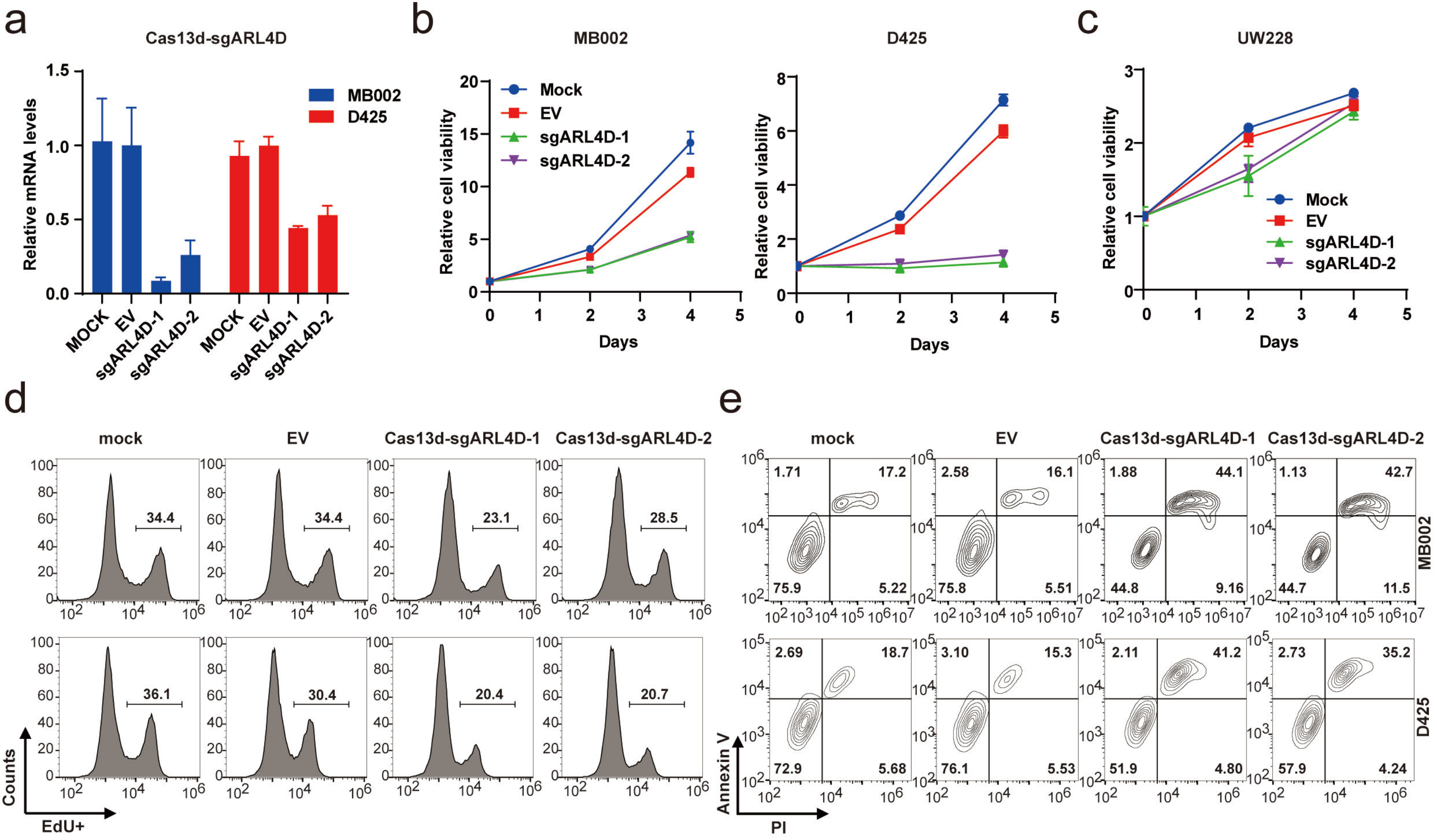

Li_et_al_Supplementary_Fig.S8

a

	MB002	D425	HDMB03	D283	D341	UW228
Number of SE-target genes	832	1099	818	1314	860	1360
Number of TE-target genes	16894	17094	13503	12335	13861	14146
ARL4D is SE-target gene	Yes	Yes	No	Yes	No	No
ARL4D rank in SE-target genes	54(6.5%)	165(15.0%)		268(20.4%)		
ARL4D rank in TE-target genes			844(6.3%)		1413(10.2%)	6492(45.9%)

b

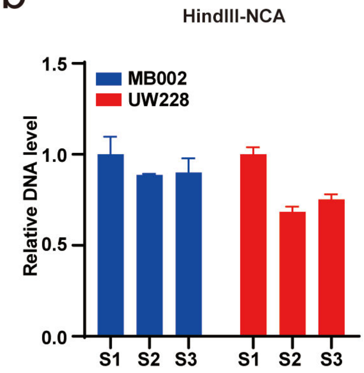

c

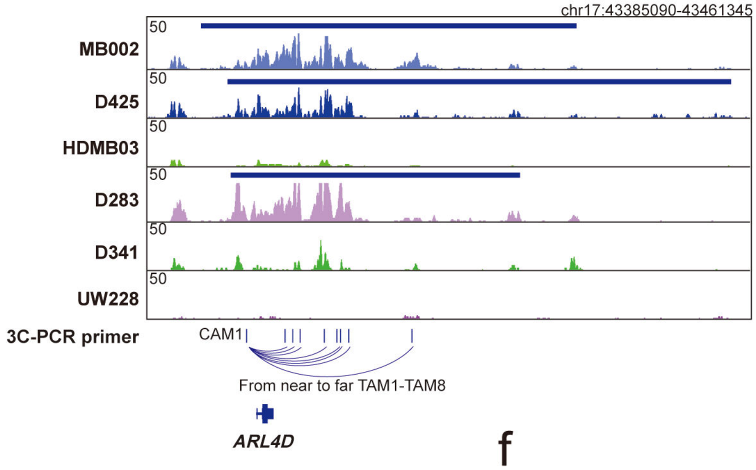

d

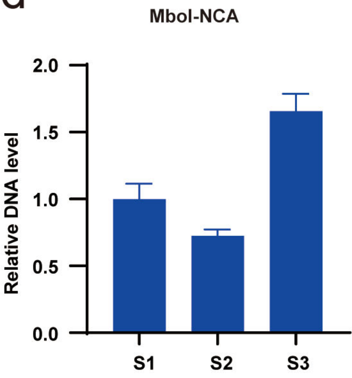

e

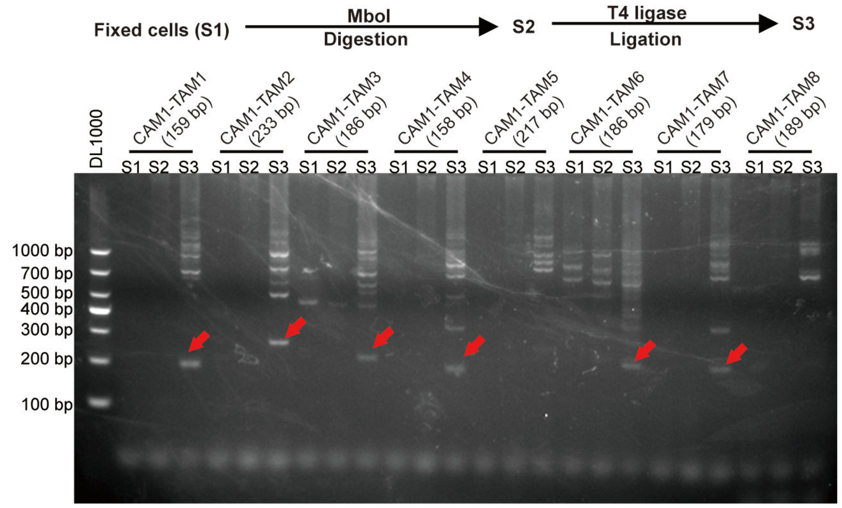

f

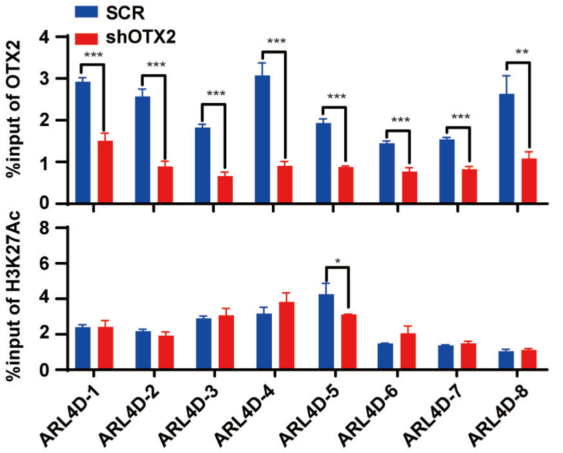

g

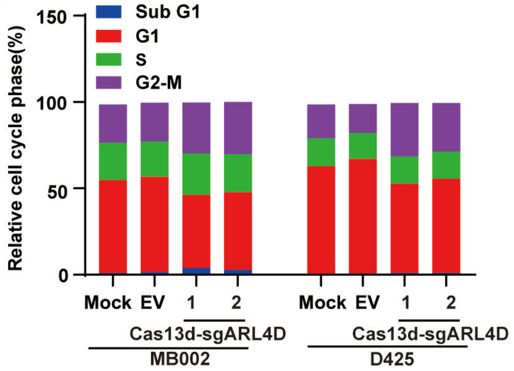

h

	Tumorsphere ratio					
	MB002			D425		
Planted cells(per well)	shSCR	shARL4D-1	shARL4D-2	shSCR	shARL4D-1	shARL4D-2
1	1/30	0/30	0/30	2/30	0/30	0/30
10	4/10	1/10	1/10	4/10	0/10	0/10
20	7/10	2/10	2/10	5/10	0/10	0/10
30	9/10	2/10	2/10	10/10	1/10	0/10
40	8/10	2/10	3/10	8/10	1/10	2/10
50	8/10	4/10	2/10	9/10	1/10	1/10
100	7/8	5/8	4/8	8/8	1/8	3/8
250	6/8	1/8	1/8	8/8	3/8	4/8
Frequencies of tumorsphere forming cells	1:38	1:218	1:254	1:19	1:545	1:355
95% Confidence Interval	26-54	130-367	147-438	14-27	259-1146	191-661
